# Bone metastases from endocrine cancer: advances in diagnosis, treatment, and prevention

**DOI:** 10.3389/fendo.2025.1669154

**Published:** 2025-12-12

**Authors:** Shuzhou Huang, Yue Shen, Hanchen Li, Tianxiang Geng, Jinhong Yan, Li Wang, Laijian Sui

**Affiliations:** 1 Department of Orthopedics, Shandong Second Medical University, Weifang, Shandong, China; 2 Department of Orthopedics, Yantai Yuhuangding Hospital, Yantai, Shandong, China; 3 Department of Orthopedics, Binzhou Medical University, Yantai, Shandong, China; 4 Department of Neurology, Affiliated Hospital of Qingdao University, Qingdao, Shandong, China

**Keywords:** endocrine cancer, bone metastasis, cross-cancer mechanism, diagnosis, treatment, prevention

## Abstract

Endocrine cancers, originating from hormone-producing organs, prefer to metastasize hematogenously to bone as a common site. Once considered rare, diagnosis rate of bone metastases presents a rising trend attribute to advanced imaging techniques and greater patient longevity. However, accurate diagnosis of bone metastases remains challenging. Diagnostic hurdles stem from heterogeneous metastatic mechanisms among cancer types and high rates of therapeutic resistance. Preventive measures remain sub-optimal, and clinical management pathways are often fragmented. Addressing these complexities requires a systematic research approach to enhance diagnostic accuracy and optimize therapeutic strategies. Present review synthesizes recent progress in the diagnosis, management, and prevention of bone metastases in endocrine tumors, emphasizing the imperative for an integrated diagnostic paradigm that leverages clinical assessment, advanced imaging, and molecular/genomic profiling to inform precision medicine. We also explore emerging hybrid predictive models that clarify the distinct biological mechanisms underpinning bone metastasis in various endocrine cancers. Ultimately, this review identifies pathways for improved therapies, refined clinical guidelines, and enhanced multidisciplinary collaboration, aiming to extend survival, improve quality of life, and ensure optimal healthcare resource utilization for patients with endocrine malignancies.

## 1 Introduction

Endocrine cancers comprise a diverse group of malignancies arising from hormone-producing tissues or hormone-dependent cells, which are typically divided into classical endocrine gland cancers, neuroendocrine tumors (NETs), and endocrine-dependent cancers (1). Among them, thyroid cancer is the most prevalent endocrine malignancies representing over 90%. Its global incidence is rising steadily, attributing to improved screening and diagnostic awareness (2, 3). The majority are well-differentiated types—papillary and follicular thyroid carcinoma—which generally have favorable outcomes but may metastasize to bone in advanced stages (4).

NETs, the second major group, originating from neuroendocrine cells dispersed throughout the gastrointestinal tract, pancreas, and lungs. The incidence of NETs is increasing worldwide, reaching approximately 1–2 cases per 100,000 individuals annually (5, 6). NETs display wide biological heterogeneity, ranging from indolent well-differentiated forms to aggressive neuroendocrine carcinomas (NECs) (1).

In addition, endocrine-dependent cancers such as hormone receptor–positive (HR^+^) breast cancer and androgen receptor–driven (AR^+^) prostate cancer are closely linked to endocrine signaling despite not arising from endocrine glands (7, 8), which is modulated by estrogen or androgen pathways, and endocrine therapy remains the cornerstone of management. However, when these tumors develop bone metastases, these endocrine-dependent cancers become markedly more aggressive, which profoundly worsens prognosis (9, 10). Although thyroid carcinomas and neuroendocrine tumors (NETs) present lower bone metastatic rates(3.9% to 4.2% in thyroid cancer and 10–15% in NETs), confirmed bone metastasis, sharply worsens the prognosis, with 10-year overall survival rates declining to 13–21% (11, 12). Skeletal metastases in endocrine malignancies cause significant morbidity through skeletal-related events (SREs), including pathological fractures, spinal cord compression, and intractable pain, which severely compromise physical function, life quality, and survival.

Notably, bone metastasis (BM) mechanisms vary by cancer type. Starting with thyroid cancer, particularly its FTC (follicular thyroid carcinoma) and MTC (medullary thyroid carcinoma) subtypes, the underlying drivers of BM remain incompletely clear but may include RET (rearranged during transfection) mutations and RAS-MAPK (mitogen-activated protein kinase) signaling abnormalities (13).For neuroendocrine neoplasms (NENs), including high-grade neuroendocrine carcinoma (NEC), BM affects 4%–15% of cases (up to 40% in carcinoids) and is linked to poor prognosis due to skeletal-related events (SREs) like pathological fractures, with its development rooted in the “seed-soil” interplay. Tumor cells (the “seed”) feature SSTR2 (somatostatin receptor 2) (supporting ^68^Ga-DOTATATE PET/CT [positron emission tomography/computed tomography] detection), BSP (bone sialoprotein) (aiding adhesion/angiogenesis), CgA (chromogranin A) (modulating the bone microenvironment), and oncogenic microRNAs (miR-210, miR-21) that promote BM (14–17). The bone microenvironment (the “soil”) is disrupted by vitamin D deficiency (from surgery, somatostatin analog [SSA] therapy, or steatorrhea), while high-grade lung NETs (neuroendocrine tumors) (e.g., SCLC [small cell lung cancer], LCNEC [large cell neuroendocrine carcinoma]) involve Hyaluronan-CD44, DKK1, and Annexin A1 pathways (18–21). Diagnosis uses multimodal imaging (^68^Ga-DOTATATE PET/CT, MRI [magnetic resonance imaging], ^18^F-FDG PET/CT), biomarkers (CgA, BSP, BSAP [bone-specific alkaline phosphatase]), and bone marrow biopsy (gold standard) (22–24), with therapies like PRRT (peptide receptor radionuclide therapy) for SSTR2-positive cases—though unknown primary tumors remain a challenge (24–28).Finally, breast cancer BM is mostly osteolytic, driven by dysregulation of the RANKL (receptor activator of nuclear factor-kappaB ligand)/OPG (osteoprotegerin) pathway, while prostate cancer BM is often osteoblastic, mediated by Wnt pathway activation (13, 29).

**Figure 1:**
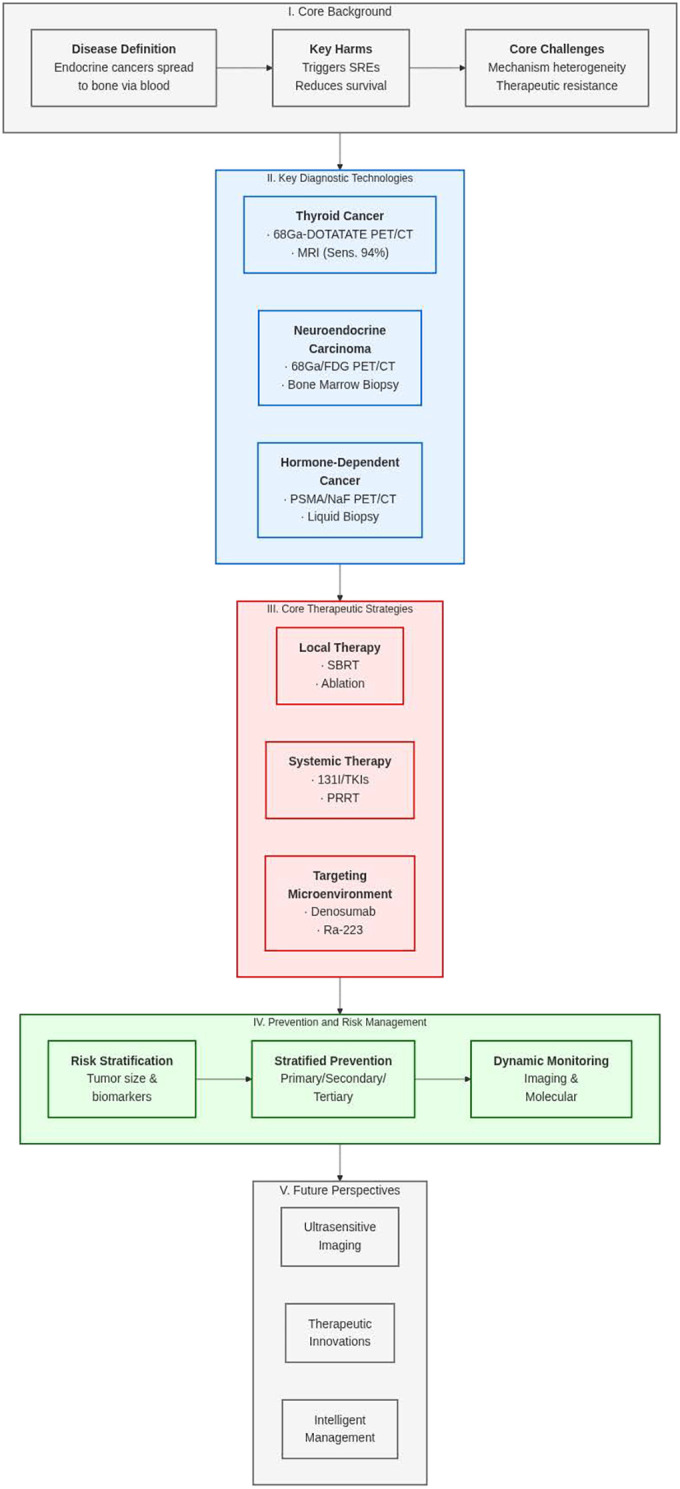
Graphical abstract.

Given these complexities, endocrine-related bone metastases present a compelling model for studying tumor–bone interactions. Translational research into key processes—such as stromal remodeling, osteotropic signaling, and tumor dormancy—offers opportunities to identify novel therapeutic targets and biomarkers. Moreover, integrating genomics, proteomics, and radiomics with clinical and imaging data can help delineate metastasis risk, enable earlier detection, and inform tailored treatment strategies. The development of multidisciplinary, individualized treatment plans—guided by molecular profiling and supported by precision medicine frameworks—holds significant promise for improving patient outcomes, reducing skeletal complications, and refining clinical management protocols. To clearly and concisely present the basic findings of this article regarding bone metastases in endocrine malignancies—including key background, diagnostic technologies, therapeutic strategies, prevention and management, and future directions—the following graphical abstract is hereby presented, as shown in **Figure 1**.

## 2 Advances in diagnostic techniques for bone metastasis of thyroid cancer

Once bone metastasis occurs in thyroid cancer, the prognosis decreases significantly, so its early and precise identification is of great clinical significance. In recent years, diagnostic techniques have advanced continuously, covering multidimensional detection means from traditional imaging to the molecular level. This article summarizes the current key technological advances in this field.

### 2.1 Clinical features and risk factors

Bone metastases in thyroid carcinoma occur predominantly in follicular thyroid carcinoma (19.2–34.0%) and poorly differentiated carcinoma (16.7–23.1%) (13) (30) (31). Patients typically present with persistent bone pain (67%), pathological fractures (23–27%), or neurological symptoms due to spinal cord compression (14–34%) (32) (33) (34). The most frequent metastatic sites include the spine (31.8–50%), pelvis, and ribs, with cervical spine lesions accounting for approximately 16% of spinal metastases (35–37). Identified risk factors for bone metastasis are primary tumor diameter greater than 4 cm (Odds Ratio = 3.2), vascular invasion (Hazard Ratio = 4.1), and the co-occurrence of BRAF(v-Raf murine sarcoma viral oncogene homolog B1) and TERT (Telomerase Reverse Transcriptase)promoter mutations (p value = 0.003) (30, 38, 39).

### 2.2 Imaging modalities for diagnosis

Structural imaging remains fundamental to diagnosis. Computed tomography (CT) demonstrates excellent sensitivity (71–100%) for detecting osteolytic lesions and enables precise mapping of cortical bone destruction, particularly through three-dimensional reconstruction in anatomically complex regions (35, 37, 40). Magnetic resonance imaging (MRI) provides high sensitivity (94%) for detecting early bone marrow infiltration. Typical findings include low signal intensity on T1-weighted imaging and high signal intensity on T2-weighted imaging with contrast enhancement, critical for evaluating spinal cord involvement (41–43). Conventional radiography is limited to detect lesions involving more than 50% cortical destruction and is primarily used for preliminary assessment of long bone fractures (42, 44).

Functional imaging further complements structural assessment. The ^99m^Tc-methylene diphosphonate (MDP) bone scan offers whole-body screening with sensitivity ranging from 56% to 95%, although its sensitivity is lower for osteolytic lesions, making it more suitable for osteoblastic metastases (40, 45, 46). The ^18^F-fluorodeoxyglucose positron emission tomography/computed tomography (^18^F-FDG PET/CT) achieves high sensitivity (92%) for detecting radioiodine (RAI)-refractory lesions but is associated with elevated costs and a false-positive rate of 15–20%. It is primarily used for systemic metastatic staging (30) (45) (47).124I-PET imaging specifically identifies RAI-avid lesions but requires thyroid-stimulating hormone (TSH) stimulation (> 30 μIU/mL), making it valuable for differentiated thyroid carcinoma (DTC) treatment planning (47, 48).^68^Ga-DOTATATE PET imaging, with a sensitivity of 98%, is specific for medullary thyroid carcinoma (MTC) and is primarily applied in neuroendocrine tumor metastasis detection (34, 48).

### 2.3 Pathological and molecular diagnostics

Histopathological examination via fine-needle aspiration or core biopsy remains the diagnostic gold standard. Follicular carcinoma typically shows vascular or capsular invasion and positive thyroglobulin (Tg) staining in 95% of cases (30, 37). Papillary carcinoma is characterized by nuclear grooves and intranuclear inclusions, with positivity for thyroid transcription factor-1 (TTF-1) (30) (49). Medullary carcinoma demonstrates calcitonin and carcinoembryonic antigen (CEA) positivity (34, 45).

Molecular diagnostics provide additional insights into disease status. Postoperative serum Tg levels exceeding 10 ng/mL are indicative of metastatic disease, with a specificity of 82% (30, 33). A high receptor activator of nuclear factor-kappaB ligand (RANKL) to osteoprotegerin (OPG) ratio (>3.5) correlates with progressive bone destruction (p = 0.01) (50). Downregulation of microRNA-124 (miR-124) is associated with an increased risk of bone metastases, with an AUC of 0.78 (39). Furthermore, combined BRAF V600E and TERT promoter mutations increase the risk of bone metastases by 5.3-fold (39, 50).

### 2.4 Diagnostic challenges and strategic approaches

Heterogeneous lesion presentation complicates diagnosis. About 34% of cases exhibit mixed osteolytic and osteoblastic features, requiring differentiation from osteosarcoma (marked by a 5-fold elevation in alkaline phosphatase) and metastatic breast cancer (estrogen receptor/progesterone receptor positivity) (35, 51, 52). Moreover, 5–20% of dedifferentiated metastatic lesions lose Tg expression. In such cases, additional markers including CD56 and PAX8 are essential for diagnosis (30, 49).

Dynamic monitoring strategies are critical for high-risk patients. Those with follicular or Hürthle cell carcinoma (FTC/HCC) should undergo whole-body bone scans or PET/CT every six months (38, 45). A Tg doubling time of less than one year strongly predicts metastatic progression (HR = 4.7) (33). Elevated C-terminal telopeptide (CTX) levels exceeding 600 pg/mL predict a 3.2-fold increased risk of skeletal-related events (SREs) (31, 53).

### 2.5 Optimization of diagnostic workflow

Patients presenting with solitary bone lesions should undergo CT or MRI localization followed by biopsy and molecular subtyping. For those with multiple lesions, whole-body PET/CT imaging is recommended prior to targeted biopsy and comprehensive genetic profiling (45, 48).

A multidisciplinary team (MDT) approach integrating endocrine surgeons, radiologists, orthopedic oncologists, and pathologists is essential. Treatment should be guided by the Spine Instability Neoplastic Score (SINS ≥ 13 indicates surgical intervention) and the Mirels score (≥ 9 suggests the need for prophylactic fixation) (37, 54–57).

## 3 Diagnostic methods and recent advances in bone metastases in neuroendocrine carcinoma

Neuroendocrine carcinoma (NEC) encompasses a heterogeneous group of malignancies arising from neuroendocrine cells, spanning well-differentiated neuroendocrine tumors (NETs) and poorly differentiated NECs, with predominant involvement of the gastroenteropancreatic tract and lung (58, 59). Bone metastasis represents a common and clinically impactful pattern of distant spread in NEC, as it not only severely impairs patients’ quality of life but also correlates with unfavorable prognosis (14, 60). Epidemiologically, bone metastasis occurs in 10%–30% of gastroenteropancreatic neuroendocrine neoplasms (GEP-NENs), while high-grade (G3) NECs present an even higher risk (≥40%) (61, 62). Notably, patients with bone metastasis exhibit a median overall survival (OS) reduced by ~50% compared to those without metastatic bone involvement (61). Given the insidious onset of symptoms in bone metastasis, timely and accurate diagnosis is critical for tailoring therapeutic strategies and improving clinical outcomes. The diagnosis of bone metastasis in NEC relies on integrating tumor biological features (e.g., differentiation grade, molecular phenotypes) with multimodal approaches including imaging, biomarkers, and histopathology. The following content details these diagnostic modalities (1).

### 3.1 The development of diagnostic techniques

The diagnosis of bone metastases in NEC relies on a multimodal approach integrating imaging modalities, biochemical biomarkers, and histopathological confirmation, with each component tailored to tumor grade, differentiation status, and clinical context. Imaging remains the cornerstone, with advanced functional techniques now dominating due to their superior sensitivity and specificity. ^68^Ga-DOTATATE PET/CT targets somatostatin receptors (SSTRs), which are highly expressed in most well-differentiated neuroendocrine neoplasms (NENs). In a study of 49 gastroenteropancreatic (GEP)-NEN patients, this modality outperformed ^18^F-FDG PET/CT in detecting bone metastases (p < 0.001) and altered staging in 12.2% of cases (14). Its advantages include high resolution, rapid acquisition, and efficacy in identifying osteoblastic lesions, though false positives may occur due to SSTR expression in inflammatory cells (60). In contrast, ^18^F-FDG PET/CT is more effective in high-grade NEC (G3 NET and NEC), where tumors exhibit increased glycolytic activity. It complements ^68^Ga-DOTATATE by detecting lesions with low SSTR expression and is particularly valuable for identifying concurrent lung or peritoneal metastases (14). For bone marrow-specific involvement—a rare but aggressive manifestation—MRI is the most sensitive (90–100%) modality for detecting early marrow infiltration and spinal cord compromise, while conventional bone scintigraphy (^99^mTc) and CT are limited by low sensitivity for small or early lesions (24, 61).

Biochemical biomarkers enhance diagnostic accuracy when combined with imaging. Chromogranin A (CGA), a secretory protein elevated in NEC patients with bone metastases, serves as a sensitive serum marker for disease progression, though it lacks specificity for bone involvement alone (60, 63). Bone Sialoprotein (BSP), an integrin ligand promoting angiogenesis and bone remodeling, correlates with bone metastasis and poor prognosis (60), while bone-specific alkaline phosphatase (BSAP)—a marker of osteoblastic activity—may be a superior prognostic indicator compared to CGA in some cohorts (64). The integration of ^68^Ga-DOTATATE imaging with CGA and BSP assays improves diagnostic accuracy (AUC > 0.75, sensitivity and specificity > 80%) compared to single modalities (60). For cases with suspected bone marrow metastasis, bone marrow biopsy remains the gold standard, key histopathological findings include nest-like distributions of small cells with scant cytoplasm, irregular nuclei, and coarse chromatin, supported by immunohistochemistry (positivity for CD56 and synaptophysin, high Ki-67 index [often >20%] for high-grade NEC) (24, 60).

Risk stratification using machine learning further refines diagnostic workflows, particularly in high-grade lung NEC. A stochastic gradient boosting (GBM) model, trained on SEER data (2010-2021), identified nine key predictors of synchronous bone metastases: age, race, sex, T/N stage, marital status, and presence of brain/liver/lung metastases. Liver metastasis emerged as the strongest predictor, with younger patients (≤65 years) with hepatic involvement facing higher risk (65). This model enables personalized imaging surveillance, directing comprehensive bone evaluation (e.g., PET/CT or MRI) to high-risk subgroups—such as patients with liver metastasis or advanced nodal disease—thereby improving early detection (65).

## 4 The evolution of bone metastasis diagnosis in endocrine-dependent cancers: from single testing to multimodal integrated strategies

Prostate cancer and breast cancer, two leading endocrine-dependent malignancies, are frequently complicated by bone metastasis in their advanced stages—notably, the incidence of bone metastasis in metastatic castration-resistant prostate cancer (mCRPC) reaches up to 90% over the disease course (66, 67), underscoring the critical need for optimized diagnostic strategies to guide precise clinical management. Below is an integrated synthesis of the core diagnostic approaches and recent advances in bone metastasis detection for these two cancers, weaving together multimodal imaging, biomarker-imaging synergy, liquid biopsy, molecular pathology, and intelligent diagnostic tools.

Imaging serves as the foundational pillar for bone metastasis diagnosis in both cancers. In prostate cancer, radionuclide bone scanning (^99^mTc-MDP) has long been a primary screening option, with a sensitivity of 62%-100% but limited specificity of 50%-80% (68). A meta-analysis of 353 patients confirmed that ^99^mTc-MDP bone scan achieves a pooled sensitivity of 67% and specificity of 88% for prostate cancer osseous metastases, far lower than MRI’s 95% sensitivity and 97% specificity (69). Osteoblastic metastases may even present as false-negative “cold zones” due to atypical radiotracer uptake. In breast cancer, plain radiographs visualize bone destruction features (e.g., osteolytic “worm-eaten” lesions) yet only reach 44-50% sensitivity, while ^99^mTc-MDP bone scanning improves whole-body detection to 85-95% but retains low specificity (60-70%). Computed tomography (CT) complements these limitations with superior anatomical resolution: In prostate cancer, CT excels at identifying osteoblastic lesions and cortical invasion but fails to detect early bone marrow infiltration. In breast cancer, multi-detector spiral CT (≤1mm slice) identifies cortical destruction with 0.5mm accuracy via 3D reconstruction, boosting osteolytic lesion sensitivity to 86% and specificity to 98%, low-dose CT (≤2 mSv) enables safe annual screening, and micro-CT achieves 20μm resolution for detecting <0.5mm microfractures.

MRI fills the gap in detecting bone marrow micrometastasis. In prostate cancer, MRI shows low T1-weighted and high T2-weighted/DWI signals, with whole-body MRI (WB-MRI) providing comprehensive skeletal coverage. In breast cancer, T1-weighted sequences combined with STIR achieve 94% sensitivity for bone marrow edema—detecting metastases 6–8 weeks earlier than overt destruction, DWI further enhances accuracy, with 91% correct malignant diagnosis when ADC <1.0 × 10⁻³ mm²/s (consistent with a 3T MRI study showing mean malignant ADC of 0.870 × 10⁻³ mm²/s) (70). WB-MRI detects multiple metastases 30% more efficiently than traditional methods via single-scan whole-skeleton coverage.

Molecular imaging has driven transformative advances, particularly with positron emission tomography (PET)-based techniques. In prostate cancer, PSMA-targeted imaging outperforms conventional modalities: ^99^mTc-PSMA SPECT/CT achieves 80% sensitivity and 100% specificity for bone metastases, detecting lesions ≤0.6cm that ^99^mTc-MDP misses, and altering management in 14.9% of patients (71). PSMA PET/CT detects microscopic metastases even at low PSA levels, with sensitivity up to 98% and specificity up to 100%, though ^18^F-PSMA-1007 may show nonspecific uptake (SUVmax>10). In breast cancer, ^18^F-FDG PET/CT quantifies metabolic activity via SUVmax, in lobular breast cancer, it reaches 93.33% specificity for bone metastases, though sensitivity is limited by sclerotic lesion hypometabolism (72). A meta-analysis of 668 breast cancer patients confirmed ^18^F-FDG PET/CT’s 93% sensitivity and 99% specificity, superior to bone scintigraphy’s 81% sensitivity (73). Novel tracer ^18^F-NaF boosts osteoblastic metastasis sensitivity to 98%, while 68Ga-PSMA PET/CT increases microscopic detection by 40% in HER2-positive cases. Dual-modality fusion (e.g., Biograph mMR MRI/PET) reduces spinal metastasis localization error from 8mm to 1.2mm.

Coupling biomarkers with imaging refines diagnostic specificity. In prostate cancer, serum PSA lacks metastasis specificity—some mCRPC patients maintain normal levels —but high PSA (>15.275ng/ml) predicts ^99^mTc-MDP-detectable bone metastases. Elevated BALP indicates active osteogenesis in osteoblastic metastases, and its dynamics reflect Ra-223 efficacy. The RANKL/OPG axis shows elevated RANKL and reduced OPG in metastatic patients, with ratios >2.5 indicating active bone destruction. In breast cancer, high-risk patients (triple-negative/HER2+/≥4 lymph nodes) screened with ^18^F-FDG PET/CT plus serum CTX achieve 96% sensitivity, ECT-positive lesions confirmed by 3.0T MRI/DWI reach 99% specificity (74). Mixed osteoblastic-osteolytic lesions are 95% confirmed via iron oxide nano-enhanced MRI plus ^18^F-NaF PET/CT. DCE-MRI Ktrans paired with PET SUVmax distinguishes response from pseudoprogression.

Liquid biopsy provides molecular context for imaging. In prostate cancer, CTC ≥5/7.5mL predicts poorer prognosis, and CXCR4^+^/αvβ3^+^ CTCs (osteo-directed phenotype) correlate with bone colonization. CTC gene expression (e.g., AR, AKR1C3) mirrors spinal metastasis profiles, enabling noninvasive phenotyping. ctDNA detects AR-V7/PTEN mutations explaining “imaging-negative” metastases. Exosomal miRNAs (miR-125a-3p, miR-330-3p) are upregulated in bone metastatic patients, with expression correlating with metastatic load (75, 76). Murine studies confirm exosomal miR-26a-5p/27a-3p suppress osteoblast mineralization, linking to osteosclerotic lesions (77). In breast cancer, CTC ≥5/7.5mL increases bone metastasis risk 4.8-fold (89% PET/CT concordance) (78). ctDNA ESR1 mutations warn of endocrine resistance 3–6 months early. Urinary NTx >600nmol/mmol creatinine raises imaging-occult metastasis risk 3.2-fold, and RANKL/OPG >2.5 correlates with PET-CT hypermetabolic volume (r=0.71) (79–81). A 134-gene CTC signature distinguishes bone-only from extra-skeletal metastases, with MAF/CAPG overexpression predicting skeletal dissemination (82).

Bone biopsy and molecular pathology remain gold standards. CT/MRI-guided biopsy differentiates osteoblastic/osteolytic prostate cancer metastases, molecular markers (ERG fusion, RUNX2, Wnt dysregulation) indicate osteo-tropic mechanisms correlatable with imaging.

Intelligent systems enhance breast cancer diagnosis. ResNet-50-based models extract 372 CT texture features for 92% accuracy, PET-CT radiomics plus ALP/CTC count constructs a nomogram (AUC = 0.91) for 5-year risk prediction, and AI platforms (e.g., IBEX) synchronize imaging and liquid biopsy data, increasing early detection by 28%.

Future directions focus on ultrasensitive detection: UCNP-SX peptide conjugates detect <100 bone marrow micrometastases via NIR-II imaging, 3D-printed microenvironment chips model tumor-bone cell interactions, explaining imaging manifestations. To synthesize the aforementioned diagnostic strategies for bone metastasis across thyroid cancer, neuroendocrine carcinoma (NEC), and endocrine-dependent cancers (prostate/breast cancer), **Table 1** presents a structured, Word-ready comparison. It systematically organizes core diagnostic methods by cancer type, summarizing key performance indicators (sensitivity/specificity), primary applicable scenarios, and inherent limitations. This integration aims to support clinicians in rapid, evidence-based diagnostic selection, catering to the precision-oriented demands of oncology practice.

**Table 1:** Word-ready comparison table of diagnostic methods applicability.

Major diagnostic category	Specific technique (abbr. + full name)	Thyroid cancer bone metastasis (TCBM)	Neuroendocrine carcinoma bone metastasis (NECBM)	Prostate cancer bone metastasis (PCBM)	Breast cancer bone metastasis (BCBM)	Key clinical tips
1. Structural Imaging	1. CT (Computed Tomography)	Sensitivity: 71 100% (osteolytic lesions), 3D reconstruction for the spine	Assists in confirming cortical destruction, requires combination with functional imaging (e.g., ^68^Ga DOTATATE)	Effective for osteoblastic lesions, unable to detect early bone marrow involvement (needs MRI supplementation)	Multi detector CT (≤1mm): 86% sensitivity (osteolytic lesions), low dose CT (≤2mSv) for annual screening	First choice for assessing cortical destruction/anatomical localization
	2. MRI (Magnetic Resonance Imaging)	Sensitivity: 94% (early bone marrow infiltration), evaluates spinal cord compression	Gold standard for bone marrow metastasis (90 100% sensitivity), ideal for G3 NEC	WB MRI covers the entire skeleton, detects micrometastases better than bone scans	T1 + STIR sequences: 94% sensitivity (bone marrow edema, detectable 6–8 weeks earlier), DWI (ADC<1.0×10⁻³mm²/s: 91% diagnostic accuracy)	First choice for detecting early bone marrow/spinal cord involvement
	3. Conventional X ray	Only detects lesions with >50% cortical destruction, used for preliminary screening of long bone fractures	Low sensitivity, only for preliminary assessment of advanced fractures	Rarely used (sensitivity <50%), alternative to CT when equipment is limited	Sensitivity: 44 50% (for osteolytic “worm eaten” lesions), only for preliminary screening	Not for confirmatory diagnosis (only a backup when CT/MRI is unavailable)
2. Functional Imaging	1. ^99^mTc MDP Bone Scan	Whole body screening (56 95% sensitivity), more effective for osteoblastic lesions	Low sensitivity (misses small lesions), used for low cost preliminary whole body screening	Traditional screening tool (62 100% sensitivity, 50 80% specificity), false negatives may occur in osteoblastic metastases	85 95% sensitivity, 60 70% specificity, positive lesions require confirmation by CT/MRI	Low cost option for whole body preliminary screening, verify positive results with precise imaging
	2. ^18^F FDG PET/CT	Detects radioiodine (RAI) refractory lesions (92% sensitivity), high cost (15 20% false positive rate)	First choice for G3 NEC (high glycolytic activity), detects lung/peritoneal metastases	Used for efficacy evaluation after RAI 223 treatment or supplementary staging in high risk patients (e.g., PSA>15ng/ml)	93% sensitivity, 99% specificity (meta-analysis), false negatives may occur in osteoblastic lesions of lobular carcinoma	Indicated for RAI refractory TCBM, G3 NECBM, and high risk BCBM
	3. ^68^Ga DOTATATE PET/CT	Only applicable for MTC (98% sensitivity)	First choice for G1/G2 NET (98% sensitivity, changes staging in 12.2% of cases)	Not applicable (no somatostatin receptor expression)	Not applicable (no somatostatin receptor expression)	Mandatory for accurate diagnosis of MTC (TCBM) and G1/G2 NET (NECBM)
	4. ^124^I PET	Only applicable for DTC (requires TSH>30μIU/mL, guides RAI treatment)	Not applicable (no iodine uptake)	Not applicable (no iodine uptake)	Not applicable (no iodine uptake)	Exclusive for pre RAI treatment assessment in DTC patients
	5. PSMA PET/CT	Not applicable (no PSMA expression)	Not applicable (no PSMA expression)	Gold standard (98% sensitivity, 100% specificity, detects lesions ≤0.6cm)	Supplementary tool for HER2 positive BCBM (detects 40% more micrometastases)	First choice for PCBM, backup for HER2 positive BCBM
	6. ^18^F NaF PET/CT	Rarely used in clinical practice	Rarely used in clinical practice	Supplementary tool for osteoblastic lesions	First choice for osteoblastic BCBM (98% sensitivity)	Preferred for BCBM with predominant osteoblastic features
3. Pathological & Molecular Diagnosis	1. FNA/Core Biopsy (Fine Needle Aspiration/Core Biopsy)	Gold standard: FTC (vascular invasion, 95% Tg+), PTC (nuclear grooves, TTF 1+), MTC (calcitonin/CEA+)	Bone marrow biopsy: small cell nests (CD56+, synaptophysin+), G3 NEC (Ki 67>20%)	CT/MRI guided biopsy, distinguishes osteoblastic/osteolytic metastases, ERG/RUNX2 detection for bone tropism mechanisms	Confirms lesion origin (ER/PR/HER2+), rules out other bone tumors (e.g., osteosarcoma)	Mandatory for confirmatory diagnosis (all cancer types)
	2. Serum Markers	Tg>10ng/mL (82% specificity), RANKL/OPG>3.5 (p=0.01), BRAF+TERT mutations (5.3× increased risk)	CGA (marker for disease progression), BSP (associated with bone metastasis and poor prognosis), BSAP (superior to CGA in some cohorts)	PSA>15.275ng/ml (predicts positive ^99^mTc MDP results), BALP (evaluates RAI 223 efficacy), RANKL/OPG>2.5 (indicates active bone destruction)	CTX>600pg/mL (3.2× increased risk of SREs), NTx>600nmol/mmol creatinine (3.2× increased risk of occult metastasis), ESR1 mutations (early warning of endocrine resistance)	Auxiliary tool (not for standalone diagnosis)
	3. Liquid Biopsy (CTC/ctDNA/exosome)	Downregulation of miR 124 (AUC = 0.78, rarely used clinically)	CTC exploration (limited to G3 NEC, immature technology)	CTC≥5/7.5mL (poor prognosis), CXCR4^+^/αvβ3^+^ CTCs (bone tropic phenotype), ctDNA (detects AR V7/PTEN mutations, explains “imaging negative” metastases)	CTC≥5/7.5mL (4.8× increased risk of bone metastasis), ctDNA ESR1 mutations (3–6 months early warning of resistance), 134 gene CTC signature (distinguishes bone only vs. extra skeletal metastases)	Used for prognostic stratification (not a routine diagnostic tool)
4. Dynamic Monitoring	1. Regular Imaging	FTC/HCC: whole body bone scan/PET/CT every 6 months, Tg doubling time <1 year (HR = 4.7, indicates metastatic progression)	G3 NEC: 18F FDG PET/CT every 3–6 months, G1/G2 NET: 68Ga DOTATATE PET/CT every 6–12 months	mCRPC: WB MRI/PSMA PET/CT every 3–6 months, PSA doubling time <3 months (high metastatic risk)	Triple negative/HER2+/≥4 lymph nodes: 18F FDG PET/CT + CTX every 6 months, bone metastasis patients: DCE MRI every 3 months (distinguishes efficacy vs. pseudoprogression)	Shorter follow up intervals for high risk patients
	2. Risk Stratification Tools	Risk factors: primary tumor >4cm (OR = 3.2), vascular invasion (HR = 4.1), BRAF+TERT mutations (p=0.003)	GBM model (SEER data): 9 predictors (liver metastasis = strongest predictor)	Combined stratification with PSA + PSA doubling time + bone scan, high risk patients (PSA>20ng/ml + doubling time <3 months) → prioritize WB MRI	AI models: ResNet 50 (92% accuracy), PET CT radiomics + ALP/CTC count (AUC = 0.91 for 5 year risk prediction), IBEX platform (28% improvement in early detection)	AI tools assist in optimizing diagnostic workflows

I. Abbreviations for cancer bone metastases

TCBM, Thyroid Cancer Bone Metastasis; NECBM, Neuroendocrine Carcinoma Bone Metastasis; PCBM, Prostate Cancer Bone Metastasis; BCBM, Breast Cancer Bone Metastasis; FTC, Follicular Thyroid Carcinoma; HCC, Hürthle Cell Carcinoma (a subtype of thyroid cancer); MTC, Medullary Thyroid Carcinoma; DTC, Differentiated Thyroid Carcinoma; NET, Neuroendocrine Tumor (well-differentiated); NEC, Neuroendocrine Carcinoma (poorly differentiated); G1/G2/G3, Tumor Grade 1/2/3 (G3 indicates high aggressiveness); mCRPC, metastatic Castration-Resistant Prostate Cancer.

II. Abbreviations for imaging examinations

CT, Computed Tomography; MRI, Magnetic Resonance Imaging; WB-MRI, Whole-Body MRI; DWI, Diffusion-Weighted Imaging (an MRI sequence); STIR, Short Tau Inversion Recovery (a fat-suppression MRI sequence); DCE-MRI, Dynamic Contrast-Enhanced MRI; PET/CT, Positron Emission Tomography/Computed Tomography; SPECT/CT, Single-Photon Emission Computed Tomography/Computed Tomography; ^99^mTc-MDP, ^99^mTechnetium-Methylene Diphosphonate (a radiotracer for bone scans); 18F-FDG, 18F-Fluorodeoxyglucose (a PET radiotracer); ^68^Ga-DOTATATE, ^68^Gallium-DOTATATE (a PET radiotracer targeting somatostatin receptors); ^124^I-PET, ^124^Iodine-PET (a PET modality exclusive to thyroid cancer); PSMA PET/CT, Prostate-Specific Membrane Antigen PET/CT (exclusive to prostate cancer); ^18^F-NaF, ^18^F-Sodium Fluoride (a PET radiotracer exclusive to osteoblastic lesions).

III. Abbreviations for pathological and molecular diagnosis

FNA, Fine-Needle Aspiration (biopsy); Tg, Thyroglobulin (a tumor marker for thyroid cancer); TTF-1, Thyroid Transcription Factor-1 (an immunohistochemical marker for thyroid cancer),CEA: Carcinoembryonic Antigen (a marker for MTC/neuroendocrine carcinoma); CGA, Chromogranin A (a marker for neuroendocrine carcinoma); BSP, Bone Sialoprotein (a marker associated with bone metastasis),BSAP: Bone-Specific Alkaline Phosphatase (a marker for osteoblastic activity); PSA, Prostate-Specific Antigen (a tumor marker for prostate cancer),BALP: Bone Alkaline Phosphatase (a marker for osteoblastic metastatic activity),CTX: C-Terminal Telopeptide (a marker for bone resorption); NTx, N-Terminal Telopeptide (a marker for bone resorption); RANKL, Receptor Activator of Nuclear Factor-KappaB Ligand (a factor promoting bone destruction)OPG: Osteoprotegerin (a factor inhibiting bone destruction); CTC, Circulating Tumor Cell; ctDNA, Circulating Tumor DNA,ER: Estrogen Receptor (a marker for breast cancer); PR, Progesterone Receptor (a marker for breast cancer); HER2, Human Epidermal Growth Factor Receptor 2 (a marker for breast cancer); ESR1, Estrogen Receptor 1 (a gene associated with endocrine resistance in breast cancer); BRAF/TERT, Oncogenes (mutations indicate high risk of TCBM); AR-V7, Androgen Receptor Splice Variant 7 (a marker for drug resistance in prostate cancer); PTEN, Phosphatase and Tensin Homolog (a tumor suppressor gene associated with prostate cancer metastasis); Ki-67, A proliferation index (higher values indicate more aggressive tumors).

IV. Abbreviations for other medical terms

RAI, Radioiodine (a treatment for differentiated thyroid cancer); SRE, Skeletal-Related Event (e.g., pathological fracture, spinal cord compression); AUC, Area Under the Curve (an indicator of diagnostic efficacy); HR, Hazard Ratio (an indicator of prognostic risk); OR, Odds Ratio (an indicator of the strength of association with risk factors); GBM, Gradient Boosting Machine (a machine learning model for risk stratification); SEER, Surveillance, Epidemiology, and End Results (a U.S. cancer statistics database); LN, Lymph Node.

## 5 Treatment of endocrine cancer bone metastases

Although conventional approaches such as surgery, radiotherapy, and bisphosphonates have achieved partial success in symptom control, long-term management remains challenging. Recent advances in understanding tumor-bone interactions have identified novel molecular targets and pathways that could be leveraged to develop more effective and specific therapies. Integrating bone-targeted agents with systemic anticancer treatments holds promise for improving clinical outcomes and minimizing skeletal-related events.

Given the substantial clinical burden and the current therapeutic limitations, optimizing the management of bone metastases is crucial for improving both survival and quality of life in patients with endocrine cancers.

### 5.1 Therapeutic strategies for bone metastases in thyroid cancer

Building upon the current understanding of molecular mechanisms, a range of therapeutic strategies has emerged and continues to evolve. Surgical management remains fundamental in treating bone metastases from differentiated thyroid cancer (DTC), especially in cases involving spinal structures. According to the American Thyroid Association (ATA) guidelines, complete resection of isolated, symptomatic metastases—particularly in patients under 45 years of age—is associated with improved survival outcomes. When radical surgery is contraindicated due to advanced age, widespread metastatic burden, or comorbidities, palliative operations such as spinal stabilization and decompression may still provide substantial symptom relief and prevent serious complications like pathological fractures and spinal cord compression.

For patients with limited metastatic burden, minimally invasive local therapies such as arterial embolization, image-guided ablation, and percutaneous vertebroplasty present valuable alternatives (83). Pre-surgical embolization is particularly useful for hypervascular lesions, helping to control intraoperative hemorrhage (84), and while it may not extend overall survival, it plays an important role in pain reduction and surgical facilitation (85, 86). Ablation approaches—including radiofrequency, microwave, and cryotherapy—are well-established for alleviating metastatic pain and reducing tumor mass (87). In vertebral involvement, percutaneous vertebroplasty under CT guidance allows for injection of polymethylmethacrylate, reinforcing vertebral stability and mitigating osteolytic discomfort (13).

External beam radiation therapy (EBRT) is a non-invasive option commonly used to manage refractory bone pain, structural instability, or neural compression (88). Although thyroid tumors are traditionally less radiosensitive, newer approaches such as stereotactic ablative radiotherapy (SABR) offer highly focused high-dose radiation with minimal exposure to adjacent critical tissues. Clinical trials report encouraging local control rates, reaching up to 88% over two years, particularly in spinal metastases (89, 90), though the long-term survival impact requires further exploration.

Radioactive iodine ^131^I therapy remains a mainstay in treating iodine-avid metastatic DTC, especially in small-volume disease where complete response may be achievable (91) (92). However, the response rate in distant metastases remains limited—only about 30–50% of patients show durable benefit—and the use of ^131^I in large skull or spine lesions requires caution due to anatomical proximity to critical neural structures (32, 93, 94). The ATA recommends repeated high-activity ^131^I therapy (3.7–7.4 GBq) guided by dosimetry to avoid marrow toxicity (45). Combined modalities, incorporating ^131^I with other treatments, have shown improved outcomes in select cases (95, 96). Other β-emitting radiopharmaceuticals, such as strontium-89 (^89^Sr) and samarium-153 (^153^Sm), may relieve metastatic bone pain, albeit with slower onset and potential hematologic side effects (13) (97). Meanwhile, α-emitter radium-223 (^223^Ra), with its potent cytotoxicity and short tissue range, is under investigation for applications beyond prostate cancer, though its role in DTC remains unclear (98).

In radioiodine-refractory DTC (RAIR-DTC), tyrosine kinase inhibitors (TKIs) have become central to systemic therapy. Among them, lenvatinib has demonstrated a significant benefit in progression-free survival (PFS) for patients with bone metastases (99), although overall survival (OS) gains remain under debate (100, 101). Apatinib, as shown in the REALITY study, has also yielded favorable outcomes in both PFS and OS metrics (102). The National Comprehensive Cancer Network (NCCN) now emphasizes the need for genomic profiling to inform TKI selection—e.g., using larotrectinib or selpercatinib for NTRK and RET gene fusions, respectively (86).

Targeting the bone microenvironment directly, bone-modifying agents such as bisphosphonates and denosumab have become standard adjuncts in managing skeletal involvement. Zoledronic acid, a potent bisphosphonate, is effective in reducing the incidence of skeletal-related events and mitigating bone pain (103), though renal toxicity limits its long-term use (104). Denosumab, a RANKL-specific monoclonal antibody, inhibits osteoclast-mediated resorption and offers an alternative with a distinct safety profile—albeit with risks such as hypocalcemia and osteonecrosis of the jaw, necessitating calcium supplementation and dental monitoring (105, 106). Importantly, sudden discontinuation of denosumab has been linked to rebound bone turnover, requiring transition strategies to alternative therapies (107). A novel agent, technetium-99 methylene diphosphonate (^99^Tc-MDP), has shown dual diagnostic and therapeutic capabilities by enhancing bone formation and suppressing osteolysis. Recent meta-analyses suggest that combining ^99^Tc-MDP with ^89^Sr improves both pain control and clinical efficacy over monotherapy (106), although further large-scale trials are warranted to confirm its long-term benefits in DTC-related bone disease.

Recent advances in thyroid cancer–related bone metastases have shifted treatment strategies from symptomatic relief toward precision-based, mechanism-guided interventions.

#### 5.1.1 Updated understanding of bone colonization

Compared to earlier emphasis on receptor activator of nuclear factor-κB ligand (RANKL)-mediated osteolysis, recent findings highlight a more complex tumor–bone interaction. Single-cell sequencing has revealed elevated expression of colony-stimulating factor 1 receptor (CSF1R) and AXL receptor tyrosine kinase in metastatic lesions (108), while tumor adhesion to vertebral endothelium is promoted by chemokine (C-X3-C motif) ligand 1/intercellular adhesion molecule-1 (CX3CL1/ICAM-1) signaling (109). Growth differentiation factor 15 (GDF15) enhances osteoblast-driven metastasis through hypoxia-inducible factor (HIF) activation (110). Clinically, a circulating tumor cell (CTC) subtype co-expressing epithelial cell adhesion molecule (EpCAM) and cluster of differentiation 44 (CD44) is linked to multifocal bone spread (111).

Local therapies have become safer and more effective. Three-dimensional (3D) navigation and intraoperative neuromonitoring improve outcomes in vertebral resection by 40%. Stereotactic body radiotherapy (SBRT) achieves 92% one-year control at 24 Gray (Gy), minimizing spinal toxicity (112, 113). Minimally invasive techniques such as radiofrequency ablation with cementoplasty offer rapid pain relief (114). Radium-223 (^223^Ra) shows promising results in thyroid bone metastases, with 79% pain relief and 64% alkaline phosphatase normalization, combination with lenvatinib enhances responses (115, 116).

#### 5.1.2 Targeted and systemic therapy expansion

Lenvatinib extends progression-free survival (PFS) to 18.3 months and accelerates pain control (98). Selpercatinib, for rearranged during transfection (RET) fusions, shows 85% objective response rate (117). Cabozantinib, targeting mesenchymal-epithelial transition factor (MET), demonstrates 31% efficacy in bone lesions (118). Immune checkpoint inhibitors combined with tyrosine kinase inhibitors (TKIs) increase CD8-positive T cell infiltration and response rates (119). Preclinical data also support chimeric antigen receptor-modified T (CAR-T) cells against thyroid-stimulating hormone receptor (TSHR) for bone-specific activity (120). Chimeric Antigen Receptor T-cell (CAR-T) therapy holds substantial promise for treating bone metastases from endocrine-related cancers (thyroid, neuroendocrine, prostate, and breast cancers), which remain refractory to conventional treatments due to tumor heterogeneity and the immunosuppressive bone microenvironment. For thyroid cancer bone metastasis (TCBM), CAR-T targeting thyroid-stimulating hormone receptor (TSHR) and rearranged during transfection (RET) is advancing: TSHR-CAR-T exhibits bone-specific activity against radioiodine-refractory DTC (RAIR-DTC) in preclinical models, while RET-CAR-T overcomes resistance to RET inhibitors in MTC bone lesions. In neuroendocrine carcinoma (NEC) and neuroendocrine tumor (NET) bone metastases, somatostatin receptor 2 (SSTR2)-CAR-T is a leading candidate, leveraging SSTR2’s high expression in G1/G2 NETs (80–90% positivity) and synergizing with peptide receptor radionuclide therapy (PRRT) to enhance bone lesion clearance (60). For prostate cancer bone metastasis (PCBM), prostate-specific membrane antigen (PSMA)-CAR-T is the most clinically mature option: phase II trials (e.g., NCT04683853) show 65% of metastatic castration-resistant prostate cancer (mCRPC) patients achieve ≥50% PSA reduction, with 21/32 patients experiencing bone lesion shrinkage (121). In breast cancer bone metastasis (BCBM), subtype-specific CAR-T strategies prevail: HER2-CAR-T benefits HER2+ cases (75% HER2 positivity in bone lesions) when combined with trastuzumab, while BCMA-CAR-T targets triple-negative breast cancer (TNBC) bone metastases, with preclinical data showing 30% bone density recovery (122). Common challenges, including immunosuppression by tumor-associated macrophages (TAMs) and bone matrix penetration barriers, are addressed via combinatorial strategies: preclinical studies show combining CAR-T with bone-modifying agents (e.g., denosumab) or immune modulators (e.g., CXCR4 antagonists) enhances CAR-T survival and infiltration in bone (123). Overall, CAR-T translation prioritizes “target precision + microenvironment modulation”: PSMA-CAR-T (prostate cancer) and SSTR2-CAR-T (NETs) are near late-phase trials, while TSHR/RET-CAR-T (thyroid cancer) and HER2/BCMA-CAR-T (breast cancer) advance through early-phase studies, aiming to transform bone metastases from intractable to manageable conditions.

#### 5.1.3 Microenvironment-targeted therapies

Bone-targeting agents remain critical. Zoledronic acid and denosumab reduce skeletal-related events (SREs) by 62% (124). Odanacatib, a cathepsin K inhibitor, promotes osteolytic lesion repair (125). Locally delivered therapies using 3D-printed scaffolds increase drug concentration 100-fold. Technetium-99 methylene diphosphonate (^99^Tc-MDP) combined with strontium-89 (^89^Sr) shows superior pain control (125).

#### 5.1.4 Precision imaging and molecular monitoring

Gallium-68–labeled prostate-specific membrane antigen (^68^Ga-PSMA) PET/CT improves radioligand targeting (126). Circulating tumor DNA (ctDNA) harboring telomerase reverse transcriptase (TERT) mutations predicts progression months in advance (127), and exosomal microRNA-222-3p (miR-222-3p) achieves 92% diagnostic accuracy (128). The National Comprehensive Cancer Network (NCCN) recommends a stepwise treatment model: thyroid-stimulating hormone (TSH) suppression and antiresorptives for asymptomatic patients, SBRT and systemic therapy for progression, and ^223^Ra with immunotherapy for extensive disease. Multidisciplinary team (MDT) involvement improves five-year survival from 27% to 44% (95).

Bone metastases show higher transforming growth factor-β (TGF-β), contributing to MET amplification. Everolimus plus denosumab delays progression (129), and TANK-binding kinase 1 (TBK1) inhibition via amlexanox restores paclitaxel sensitivity (130). Prognostic models integrating PET parameters, ctDNA, and bone biomarkers achieve an area under the curve (AUC) of 0.91 (121). CTCs ≥5/7.5 mL triple the SRE risk (131).Emerging approaches include bispecific antibodies targeting prostate-specific membrane antigen (PSMA) and cluster of differentiation 3 (CD3), which increase CD8-positive T cells 9-fold (132), and clustered regularly interspaced short palindromic repeats (CRISPR)-mediated sodium-iodide symporter (NIS) gene reactivation for radioiodine sensitization (124). Artificial intelligence models analyzing CT scans reach 94% accuracy in predicting bone lesion pathology (128).

In conclusion, the therapeutic landscape for thyroid cancer bone metastasis is deeply intertwined with its underlying biology. A multimodal approach—encompassing surgery, interventional radiology, radiotherapy, and systemic or targeted therapies—should be tailored according to individual tumor burden, anatomical risk, and molecular profile. Continued elucidation of tumor–bone interactions is crucial for optimizing patient outcomes and advancing precision medicine in this challenging clinical context.

### 5.2 Therapeutic strategies and emerging advances in neuroendocrine carcinomas

Neuroendocrine carcinomas (NECs) constitute a diverse category of malignancies characterized by variable degrees of differentiation and grading (G1–G3), leading to marked heterogeneity in biological behavior and clinical aggressiveness. At initial presentation, a large proportion of patients exhibit distant metastases—most frequently involving the liver, bone, or lungs—which substantially diminish median survival and contribute to morbidity through skeletal-related complications (133, 134). Historically, therapeutic management was dominated by conventional cytotoxic chemotherapy, such as cisplatin–etoposide combinations, particularly for high-grade NECs. However, these regimens were often constrained by severe toxicity and limited long-term efficacy. For well-differentiated subtypes, somatostatin analogs (SSAs) were employed mainly for symptomatic control, with minimal impact on disease progression (24, 135). In recent years, the therapeutic landscape of NECs has undergone a major shift toward biologically driven and individualized treatment strategies. Innovations such as peptide receptor radionuclide therapy (PRRT) for somatostatin receptor–positive tumors, mTOR inhibitors (e.g., everolimus), and advanced diagnostic tools integrating biomarkers (e.g., chromogranin A) with functional imaging (e.g., ^68^Ga-DOTATATE PET/CT) have collectively advanced precision oncology in this field (14). The following section summarizes evidence-based treatment approaches stratified by NEC subtype and metastatic burden, with emphasis on emerging modalities addressing persistent therapeutic challenges.

#### 5.2.1 Systemic therapies

Systemic therapies represent the cornerstone of management for disseminated bone metastases, aiming to suppress tumor growth while modulating interactions within the bone microenvironment. Chemotherapy remains the first-line regimen for high-grade neuroendocrine carcinomas (NECs), including small-cell lung NEC (SCLC), large-cell lung NEC (LCNEC), and grade 3 gastroenteropancreatic neuroendocrine neoplasms (GEP-NENs). Platinum-based combinations—typically cisplatin with etoposide—have shown notable activity against metabolically active disease (61, 62).However, treatment feasibility may be limited by systemic toxicity and patient tolerance. For instance, in a patient with NEC and diffuse bone marrow metastases (ECOG 3–4), cisplatin/etoposide was contraindicated due to severe cytopenias (hemoglobin 29 g/L, platelets 49 × 10⁹ /L) (24, 135). In contrast, well-differentiated G1/G2 neuroendocrine tumors (NETs) with bone involvement often respond to less toxic regimens such as capecitabine combined with temozolomide, achieving disease stabilization in low-grade skeletal disease (136, 137).

Somatostatin analogs (SSAs), including octreotide and lanreotide, are central to controlling hormone-related symptoms (e.g., flushing, diarrhea) and exert antiproliferative effects in low-grade NETs (135, 138). Current consensus guidelines recommend SSAs as first-line therapy for G1/G2 NETs with bone metastases, especially in patients with confirmed somatostatin receptor (SSTR) expression by functional imaging (61, 135).Beyond symptom control, peptide receptor radionuclide therapy (PRRT) has emerged as a transformative modality for SSTR-positive tumors, delivering targeted cytotoxicity through radiolabeled ligands such as ^177^Lu-DOTATATE or ^90^Y-DOTATOC. Clinical studies have demonstrated tumor regression in up to 47% of GEP-NETs and sustained symptom improvement, with one case achieving 27 months of progression-free survival following PRRT (60, 139–141). Nevertheless, limited accessibility in certain regions continues to restrict its broader use (24, 135).

Molecularly targeted therapies further expand systemic options by disrupting signaling pathways involved in tumor–bone crosstalk. The mammalian target of rapamycin (mTOR) inhibitor everolimus prolonged progression-free survival in advanced non-functional NETs, including cases with osseous metastases, as shown in the RADIANT-4 trial (24, 135) (24, 142). Yet, clinical outcomes remain variable: in a patient with bone marrow–infiltrative NEC, everolimus (10 mg/day) induced leukopenia and progressive disease, reflecting limited efficacy in aggressive subtypes (24, 135). Similarly, anti-angiogenic therapy with sunitinib has demonstrated benefit in pancreatic NETs with bone lesions by targeting the hypervascular bone niche (138, 143).For metaiodobenzylguanidine (MIBG)-avid tumors, such as pheochromocytomas and paragangliomas, ¹³¹I-MIBG therapy yields symptom relief in more than half of treated patients, though bone marrow suppression remains a dose-limiting adverse event (61, 144).Emerging strategies involve immune checkpoint inhibitors (ICIs), designed to restore antitumor immunity within the immunosuppressive bone milieu. Early-phase studies combining ICIs with chemotherapy, radiotherapy, or anti-angiogenic agents have shown encouraging signals of efficacy in high-grade NECs with skeletal metastases (65, 145). Moreover, novel nanomaterial-based drug delivery systems—such as fucoidan-coated magnetic nanoparticles and biomimetic magnetosomes—are being investigated to enhance ICI delivery, increase tumor specificity, and minimize systemic toxicity (65, 146, 147).

#### 5.2.2 Local therapeutic interventions

Local modalities complement systemic approaches by addressing focal skeletal disease and preventing skeletal-related events (SREs). Palliative radiotherapy remains highly effective for intractable bone pain and spinal cord compression, two major sources of morbidity in patients with bone metastases. Evidence from randomized trials confirms that a single 8 Gy fraction provides pain control equivalent to multi-fraction schedules (e.g., 20 Gy in 5 fractions) while reducing treatment burden (139, 148, 149). In a cohort of patients with neuroendocrine neoplasms (NENs) and bone metastases, 34.1% underwent palliative radiotherapy, which correlated with improved quality of life and decreased opioid use [4].Surgical management is reserved for selected indications, including pathological fractures, spinal instability, or isolated lesions with curative potential. Although surgery is performed in only ~2% of NEN bone metastasis cases, it is critical for preventing irreversible neurologic deficits due to cord compression (134, 150, 151). En-bloc resection of solitary bone metastases in well-differentiated NETs has yielded durable local control, provided that patient selection appropriately balances operative risk against therapeutic benefit (61, 151).

#### 5.2.3 Supportive care and SRE prophylaxis

Supportive management remains essential to minimize therapy-related toxicity and reduce SRE incidence. Bone-modifying agents (BMAs)—such as bisphosphonates (e.g., zoledronic acid) and the RANKL inhibitor denosumab—constitute the foundation of SRE prevention by suppressing osteoclast-driven bone resorption. A meta-analysis of randomized trials demonstrated that denosumab delayed the time to first SRE more effectively than bisphosphonates in patients with solid tumor bone metastases, including those with NENs (134, 152). In practice, BMAs are recommended for patients with extensive skeletal disease, rapid progression, or high-grade histology, ideally initiated within 7–14 months after bone metastasis diagnosis to prevent early complications (134).Pain management should follow the World Health Organization (WHO) analgesic ladder: paracetamol and non-steroidal anti-inflammatory drugs (NSAIDs) for mild pain, and opioids for more severe symptoms. In a study of 102 patients with NEN bone metastases, 43.5% achieved satisfactory pain control using first-line analgesics, emphasizing the importance of timely intervention (134). For patients with severe cytopenias caused by bone marrow infiltration, supportive measures such as blood transfusions and hematopoietic growth factors (e.g., interleukin-11 for thrombocytopenia) are indispensable to maintain treatment feasibility (24, 135). Additional supportive strategies include nutritional supplementation to address cancer-related cachexia and structured physical therapy to preserve mobility and prevent falls—key to reducing secondary fractures and maintaining quality of life (24, 135).

In summary, the management of bone metastases in NECs and NENs requires a comprehensive, multidisciplinary framework that integrates systemic, local, and supportive interventions. Established treatments—including platinum-based chemotherapy, peptide receptor radionuclide therapy, and bone-modifying agents—have significantly improved outcomes. Nonetheless, challenges persist, such as limited evidence for immunotherapy efficacy and the absence of standardized PRRT dosing strategies in this population. Future research should emphasize precision oncology approaches, including machine learning–based stratification models to identify patients at greatest risk for skeletal complications (65), thereby enabling individualized treatment and minimizing unnecessary interventions.

### 5.3 Comprehensive treatment strategies and latest advances in bone metastases of endocrine-dependent cancers

Bone metastasis represents a defining feature of advanced endocrine-dependent cancers, specifically prostate cancer (PCa) and breast cancer (BCa), which severely compromises bone homeostasis and skeletal integrity, thereby posing immense challenges to clinical management. The therapeutic paradigm for these metastases has shifted dramatically from single-modality interventions to multimodal, precision-driven strategies—shaped by insights into core mechanisms like Paget’s “seed and soil” theory, epithelial–mesenchymal transition (EMT), and the tumor-bone “vicious cycle.”

Bone metastases in endocrine-dependent malignancies such as prostate and breast cancer represent a pivotal stage of disease progression and a major cause of morbidity. Despite distinct biological profiles—osteoblastic predominance in prostate cancer and osteolytic features in breast cancer—both share critical molecular mechanisms involving epithelial–mesenchymal transition (EMT), chemokine-driven bone homing, and reciprocal tumor–bone interactions that generate a self-sustaining “vicious cycle” of osteolysis and osteogenesis (153–157). Management has therefore evolved toward a multimodal, mechanism-oriented strategy combining endocrine, cytotoxic, targeted, immune, and bone-modifying therapies, supplemented by precision local interventions and multidisciplinary care.

#### 5.3.1 Systemic and endocrine-based therapies

Endocrine manipulation remains the foundation of systemic management. In prostate cancer, androgen deprivation therapy (ADT) through gonadotropin-releas183ing hormone (GnRH) agonists or antagonists and androgen receptor (AR) inhibitors such as apalutamide and enzalutamide improves overall survival (158–160). The CYP17 inhibitor abiraterone extends benefit in metastatic castration-resistant prostate cancer (mCRPC) (161, 162). Similarly, in hormone receptor-positive (HR^+^)/HER2-negative breast cancer, aromatase inhibitors (AIs) and selective estrogen receptor degraders (SERDs) such as fulvestrant remain first-line, often combined with cyclin-dependent kinase 4/6 (CDK4/6) inhibitors to overcome resistance (159, 160). New-generation SERDs like elacestrant have demonstrated activity even after CDK4/6 inhibitor failure (153, 154). Targeting the PI3K/AKT/mTOR pathway, particularly in PIK3CA-mutant disease, has yielded additional benefit (163, 164).

#### 5.3.2 Chemotherapy and targeted approaches

Chemotherapy remains an essential component for endocrine-refractory or rapidly progressive disease. In prostate cancer, docetaxel improves survival in both hormone-sensitive and resistant settings, while cabazitaxel is reserved for docetaxel-refractory cases (165). In breast cancer, sequential single-agent regimens are preferred for bone-only or indolent disease, reserving combination therapy for visceral crisis (160). Targeted therapies such as PARP inhibitors (e.g., olaparib) benefit patients harboring BRCA1/2 or ATM mutations (165), and their combination with endocrine or AR blockade has reduced progression risk (166). HER2-positive breast cancer patients derive major survival advantages from trastuzumab- and pertuzumab-based regimens, often combined with taxanes (160, 167, 168).

#### 5.3.3 Bone-modifying agents and microenvironment modulation

Bone-targeted therapy constitutes a shared therapeutic axis across both malignancies. Bisphosphonates (e.g., zoledronic acid, ibandronate) and the monoclonal antibody denosumab—an inhibitor of receptor activator of nuclear factor-κB ligand (RANKL)—effectively reduce skeletal-related events (SREs) (160, 169–177). Denosumab shows superiority in SRE prevention, though monitoring for osteonecrosis of the jaw and hypocalcemia remains critical (178). Recent refinements, including liposomal zoledronic acid and conjugated forms such as zoledronic acid–paclitaxel (ZOL-PTX), enhance drug targeting and limit nephrotoxicity (179). Moreover, dual inhibition of RANKL and PI3K/AKT/mTOR signaling further prolongs skeletal protection (180, 181).

Interventions targeting the bone niche—such as CXCR4 antagonists (plerixafor), integrin αvβ3 inhibitors, and hypoxia-inducible factor-1α (HIF-1α) modulators—disrupt metastatic colonization and enhance chemotherapy response (182, 183). Anti-sclerostin antibody romosozumab and sustained-release bone morphogenetic protein 2 (BMP-2) systems promote bone formation and mitigate osteolysis (184). These approaches reflect a growing emphasis on restoring bone homeostasis alongside tumor control.

#### 5.3.4 Radiotherapy, radiopharmaceuticals, and surgery

Radiotherapy remains integral for pain relief and local control. External beam radiation therapy (EBRT), intensity-modulated radiation therapy (IMRT), and stereotactic body radiotherapy (SBRT) achieve rapid analgesia and high precision (161, 177, 185–193). Radionuclide therapies such as strontium-89 and radium-223 provide systemic skeletal targeting, the combination of Ra-223 with abir (121). Lutetium-177- and actinium-225-labeled PSMA ligands have shown remarkable efficacy in mCRPC, achieving substantial prostate-specific antigen (PSA) declines (194). Surgical intervention remains indispensable for pathological fractures, spinal cord compression, or structural instability, with decision frameworks including Mirels score and SINS guiding management (54, 55, 192, 193, 195–203).

#### 5.3.5 Immunotherapy, nanotechnology, and emerging modalities

Immunotherapy is gaining traction in bone-predominant disease. PD-1 blockade with pembrolizumab and CTLA-4 inhibition with ipilimumab have shown synergistic activity, increasing CD8^+^ infiltration and improving lesion control (204). Novel cell-based therapies, including PSMA-targeted and RANKL-directed CAR-T cells, as well as IL-15-enhanced γδ T cells, significantly reduce osteolytic activity in preclinical models (205, 206). Parallel innovations in nanomedicine—such as folate-linked liposomal mitoxantrone and hydroxyapatite-bound doxorubicin—achieve superior bone accumulation and lower systemic toxicity (182, 183). Thermal ablation using magnetic nanoparticles and bisphosphonate–denosumab–Ra-223 sequences further enhance pain control and local suppression (207–210). Recent advances in nanomedicine are paving the way for a new era of targeted therapy in endocrine cancer–related bone metastases. Bone-targeted nanoparticles, including hydroxyapatite-bound doxorubicin (DOX) and folate-linked liposomal mitoxantrone, have demonstrated superior localization within the mineralized bone matrix, achieving higher intralesional drug concentrations while minimizing systemic exposure and toxicity (211, 212). Folate receptor-mediated liposomal delivery enhances selective uptake by tumor cells, particularly in hormone-dependent cancers such as breast and prostate carcinoma, improving therapeutic efficacy and safety profiles (213). Hydroxyapatite-based nanocarriers exploit strong affinity for bone tissue, acting as dual-function systems that both release chemotherapeutic agents and support bone regeneration. Moreover, magnetic nanoparticles are being developed for localized hyperthermia or thermoablative therapy, showing promise in controlling pain and suppressing tumor growth within osseous lesions (214). Integration of these nanotherapeutic platforms with current bone-modifying agents—such as bisphosphonates, denosumab, or the α-emitter radium-223—may further enhance skeletal protection and therapeutic outcomes (211). Despite challenges in large-scale production, biodistribution variability, and long-term biocompatibility, these nanomedicine strategies represent compelling candidates for early-phase clinical trials targeting bone-dominant metastases, offering the potential to reshape standard treatment paradigms for endocrine malignancies with skeletal involvement.

#### 5.3.6 Metabolic and epigenetic reprogramming

Targeting tumor metabolism has introduced new therapeutic angles. Pyruvate dehydrogenase kinase (PDK) inhibition with dichloroacetate reverses the Warburg effect, while glutaminase inhibitors (e.g., CB-839) synergize with bisphosphonates to reduce osteoclastogenesis (215, 216). Epigenetic regulators such as EZH2 inhibitors (tazemetostat) and DNA methyltransferase inhibitors (decitabine) potentiate AR or endocrine blockade responses (217). Complementary interventions, including curcumin nanoparticles, triptolide derivatives, and microbiota modulation, further modulate immune-metabolic equilibrium (183, 218–222).

#### 5.3.7 Precision monitoring and multidisciplinary integration

**Figure 2:**
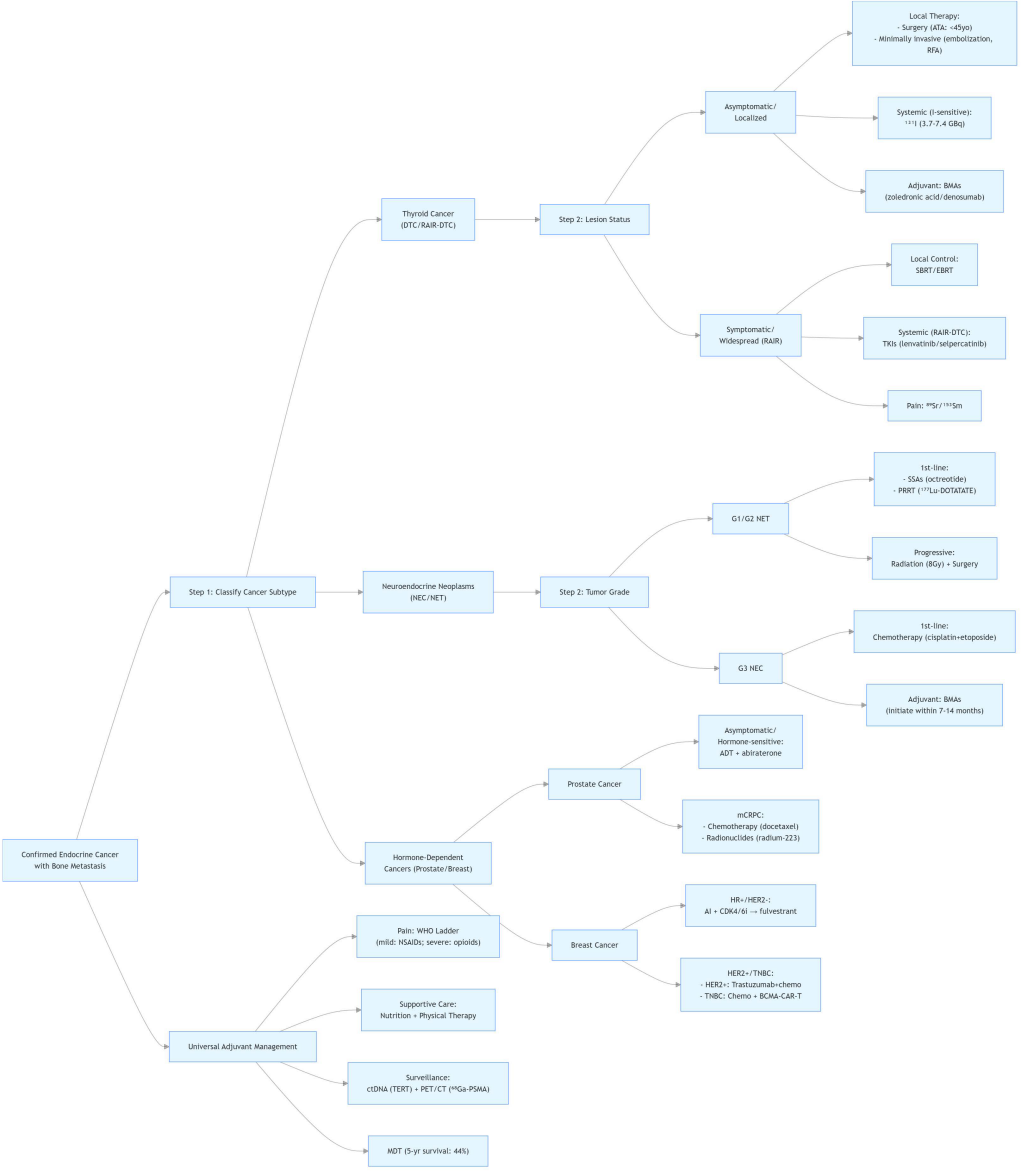
Stratified treatment flowchart for endocrine cancers with bone metastases.

Advances in imaging and molecular diagnostics have improved early detection and therapeutic precision. 18F-NaF PET/CT offers superior resolution for microlesions (208, 223), while circulating tumor cells (CTCs) and ctDNA analyses enable early assessment of bone metastatic activity and treatment response (210, 224). Artificial intelligence-based prediction models outperform conventional scoring systems in fracture risk evaluation (225, 226). Multi-omic profiling—including identification of COL11A1^+^ fibroblasts and elevated circulating progastrin (hPG80)—refines prognostic stratification (219, 227–229). A multidisciplinary team (MDT) approach integrating urology, oncology, radiology, and nuclear medicine ensures individualized, evidence-based care (230, 231). To present more intuitively the stratified treatment logic and specific implementation pathways for endocrine cancers with bone metastases (including thyroid cancer, neuroendocrine carcinoma, and endocrine-dependent cancer) developed based on cancer type, disease progression, and molecular characteristics, the corresponding stratified treatment flowchart (**Figure 2**) is hereby attached.

## 6 Significance of preventing bone metastases in endocrine

As established bone lesions are often refractory to treatment, early intervention and prevention strategies are critical. Targeting molecular mechanisms that drive bone colonization may delay metastasis onset and improve survival. Therefore, focusing on the prevention of skeletal involvement is essential to enhance long-term disease control and optimize patient care in endocrine oncology.

### 6.1 Preventive strategies for bone metastasis in thyroid cancer: integrating recent advances in precision medicine

With the continuous advancements in comprehensive treatment and precision medicine for thyroid cancer, preventive strategies against bone metastasis have become more targeted and individualized. Early detection and intervention play a critical role in preventing the progression to advanced stages. For patients with high-risk sites, such as the spine and femur, early screening and intervention are essential. The use of 3D navigation and intraoperative nerve monitoring during surgeries significantly enhances safety, allowing for effective control of small bone lesions before progressing to more invasive procedures like spinal resection (30, 109, 112). Stereotactic body radiation therapy (SBRT) has shown remarkable efficacy in increasing local control rates while minimizing spinal cord exposure, making it a preferred approach for early-stage bone lesions (41, 113, 232). Furthermore, radiofrequency ablation combined with bone cementoplasty has proven effective in preventing pathological fractures by stabilizing bone structure in the early stages (233).

Systemic preventive strategies are critical for high-risk patients. Early administration of targeted therapies and immune treatments can delay or even reverse the development of bone metastasis. Drugs like lenvatinib and selpercatinib target tumor biology to reduce bone invasion (98, 118, 234). Immune therapy combined with targeted treatments enhances CD8+ T-cell infiltration, thereby boosting immune surveillance and preventing the formation of bone metastasis (235–237). Bone-modifying agents such as bisphosphonates and denosumab also play an important role in reducing the risk of bone-related events and protecting bone integrity (124, 125, 238).

Risk stratification and early warning systems are crucial for enhancing the precision of preventive measures. A deep learning-based model, integrating parameters such as ^18^F-FDG PET/CT metabolic activity (SUVmax>8.2), ctDNA mutations in the TERT promoter, and bone metabolic markers (ALP >120 U/L), provides a reliable tool for predicting bone-related events (127, 239). Liquid biopsy assays that detect specific miRNA combinations (such as miR-146b, miR-222) have demonstrated an impressive sensitivity of 94%, enabling early detection of micro-metastasis (240).

Molecular targeting and microenvironment modulation have emerged as promising strategies in the prevention of bone metastasis. Early intervention in the RANKL pathway with denosumab (120 mg/month) has shown to reduce the incidence of bone metastasis (175, 241). TGF-β signaling inhibitors and histone deacetylase inhibitors have demonstrated potential in preclinical models to reduce the tumor burden of bone metastasis and inhibit angiogenesis (129, 242–244). Additionally, CXCR4 antagonists have been shown to reduce myeloid-derived suppressor cells (MDSCs) in the bone marrow, enhancing CD8+ T-cell infiltration and preventing the establishment of a bone metastatic niche (245).

Innovative technologies have significantly enhanced the ability to detect and prevent bone metastasis at early stages. Molecular imaging techniques, such as ^68^Ga-PSMA PET/CT, combined with 177Lu therapy, allow for enhanced tumor localization and precision targeting, thus offering a powerful tool for early intervention (126, 246). Nanoparticle-based drug delivery systems, such as β3 integrin-targeted hydroxyapatite nanoparticles, improve drug concentration in bone metastatic lesions, enabling more effective local therapy (247, 248). Moreover, CRISPR/Cas9-mediated gene silencing, such as NKX2–8 knockdown, has been shown to reduce bone metastasis formation by 73%, offering a novel preventive strategy (108, 237).

The three-level prevention system has been established to guide clinical practice across different stages of bone metastasis. For patients at high risk but without detectable metastasis, primary prevention with TSH suppression therapy combined with bone-protective agents has been shown to reduce the incidence of bone metastasis by 41% over five years (45, 95). In patients with oligometastatic disease, SBRT combined with targeted therapy has improved progression-free survival (PFS) to 68% over two years (112). For patients with extensive bone metastasis, a combination of 223Ra and immune checkpoint inhibitors has extended median survival by 4.3 months (115).

In terms of complications, denosumab has been shown to reduce hypercalcemia more rapidly than bisphosphonates, providing a more immediate therapeutic benefit (249). In cases of pathological fractures, the combination of 3D-printed titanium alloy prostheses and postoperative radiotherapy has reduced mechanical complications by 58% (250). Moreover, for spinal cord compression, vertebroplasty combined with 125I seed implantation has demonstrated a pain relief rate of 94% (251).

Future research will focus on further optimizing the prevention of bone metastasis through several innovative avenues. Developing humanized PDX models that preserve the immune microenvironment for drug screening (252), designing inhibitors targeting epigenetic markers specific to bone metastasis (such as PCAT7) (253), and optimizing the timing and sequencing of multi-modal therapies (e.g., combining radiation with immunotherapy to enhance antitumor effects) (109) will be key areas of development. Additionally, exploring circadian rhythm regulation mechanisms, such as the role of BMAL1 gene knockout in accelerating bone metastasis, may open new therapeutic windows in time-based medicine.

### 6.2 Preventive strategies for bone metastases in neuroendocrine neoplasms

Preventive strategies for bone metastases (BMs) in neuroendocrine neoplasms (NENs)—including neuroendocrine carcinoma (NEC)—focus on early detection, risk stratification, and bone health preservation to reduce skeletal-related events (SREs) and improve survival and quality of life. Early detection relies on advanced imaging. ^68^Ga-DOTATATE PET/CT shows high sensitivity (85%–95%) for somatostatin receptor (SSTR)-positive BMs, outperforming bone scintigraphy and CT (14, 60, 254). In high-grade (G2/G3) NEC, dual-tracer imaging (^68^Ga-DOTATATE and ^18^F-FDG PET/CT) detects asymptomatic lesions, refines staging, and alters management in 12.2%–14.3% of patients, redirecting care toward preventive interventions (14, 60). MRI provides 90%–100% sensitivity for bone marrow infiltration and spinal cord involvement, particularly in patients with unexplained anemia, thrombocytopenia, or elevated lactate dehydrogenase (LDH) (14, 60, 255).

Biochemical surveillance complements imaging. Chromogranin A (CgA), with sensitivity up to 81%, reflects metastatic burden but lacks bone specificity[8]. Bone sialoprotein (BSP) correlates with BM presence and poor prognosis, while bone-specific alkaline phosphatase (BSAP) offers better prognostic value for adverse outcomes (14, 60, 62). Additional markers such as type I collagen propeptide (PINP) and N-telopeptide (NTx) indicate bone metabolism. Combined use of ^68^Ga-DOTATATE imaging, CgA, and BSP enhances diagnostic accuracy (AUC > 0.75, sensitivity and specificity > 80%) (60). Elevated inflammatory markers such as high-sensitivity C-reactive protein (hs-CRP) and alkaline phosphatase (ALP) may also signal early BM in unknown primary NEC (60).

Risk stratification directs preventive efforts. Patients with liver or lung metastases have markedly increased BM risk (odds ratio [OR] = 32.98 and 35.78, respectively) (60, 256), thus, ^68^Ga-DOTATATE PET/CT screening is recommended. In high-grade lung NECs, machine learning models (e.g., gradient boosting, AUC 0.723) integrate clinical and metastatic factors for individualized risk prediction. SHapley Additive exPlanations (SHAP) analyses highlight liver metastasis, nodal stage, age, and sex as key predictors, guiding surveillance priorities (257). For hereditary syndromes such as multiple endocrine neoplasia type 1 (MEN1), regular screening remains vital since primary hyperparathyroidism accelerates bone loss and fracture risk (257).

Bone health management involves correcting vitamin D deficiency, which affects 46%–81% of NEN patients. Supplementation (cholecalciferol 1000–2000 IU/day, serum 25(OH)D >30 ng/mL) preserves bone mineral density and may improve outcomes (19, 255). Bone-modifying agents (BMAs), including bisphosphonates and denosumab, are considered for patients with multiple BMs, rapid progression, or high-grade tumors. Data from breast and prostate cancer show BMAs delay SREs, with denosumab offering superior fracture protection (14, 258). In MEN1-related hyperparathyroidism, parathyroidectomy or calcimimetics (e.g., cinacalcet) mitigate bone loss and skeletal complications (254, 256).

Lifestyle and follow-up are complementary. Adequate calcium and protein intake, low-impact exercise (e.g., walking, swimming), and avoidance of bone trauma maintain skeletal strength. Routine monitoring every 3–6 months with imaging, biomarkers, and clinical evaluation ensures timely adjustment of preventive measures, rising CgA or BSP may prompt early initiation of BMAs (60, 62). Patients with prior SREs require closer monitoring (every 2–3 months) as the median time to a second SRE is 10 months (259).

Despite progress, preventive care still faces gaps—particularly the lack of prospective trials on BMAs for SRE prevention in NENs and the need for BM-specific biomarkers. Future research should refine risk models by incorporating molecular data (e.g., Ki-67, TP53/RB1 mutations) and explore novel tracers (e.g., SSTR5 ligands) to improve detection. Studies combining vitamin D and BMAs in high-risk patients may further reduce BM-associated morbidity and mortality.

### 6.3 Strategies and advances in the prevention of prostate cancer bone metastases

Effective prevention of bone metastasis in prostate cancer requires integration of emerging therapeutic insights with proactive strategies targeting the tumor-bone microenvironment, immune evasion, hormonal axis alterations, and epigenetic dysregulation. Here, we systematically align prevention approaches with current therapeutic strategies, aiming to establish a scientifically rigorous framework for translational application.

Modulation of the bone microenvironment is critical. Prophylactic administration of denosumab (120 mg monthly) significantly reduced skeletal metastasis incidence by 42% in high-risk patients (260). Third-generation bisphosphonates, particularly when combined with TGF-β inhibitors, provide dual regulation of osteoclastic and osteoblastic activities (166, 261), preserving skeletal integrity. Maintenance of serum vitamin D3 levels above 30 ng/mL inhibits EMT processes via CaSR activation (262, 263). Furthermore, mechanical stimulation at 30 Hz enhances connexin43-mediated intercellular communication among osteocytes, decreasing secretion of the pro-metastatic factor OPN by 41% (264).

Inhibiting chemokine signaling pathways is another essential preventive approach. Periodic pulsed administration of CXCR4 inhibitors, such as plerixafor, eradicates dormant tumor cells within the bone marrow niche (265). Liquid biopsy detection of KLF4/RUNX2 ratios (AUC = 0.87) and exosomal miR-181a-5p levels can predict bone metastasis risk up to eight weeks earlier than conventional imaging (266, 267), enabling timely preventive interventions.

Optimization of hormonal pathways also supports metastasis prevention. Early identification of DNA repair deficiencies (e.g., BRCA1/2, ATM mutations) facilitates targeted prophylactic use of PARP inhibitors (e.g., olaparib) in combination with abiraterone, reducing radiological progression risk by 34% (268). The development of dual CYP17A1/CYP11B2 inhibitors offers a future avenue for simultaneous blockade of androgen and aldosterone synthesis (121).

Immune-based prevention has gained momentum. Neoantigen vaccines such as NeoVax induce durable T-cell responses in MSI-H patients, achieving an 82% five-year metastasis-free survival (269). Modulation of the gut microbiota with Akkermansia muciniphila enhances bone marrow IFN-γ+ CD8+ T-cell populations by 38%, suppressing osteoclast precursor differentiation (253).

Advances in targeted delivery systems provide additional preventive avenues. Hydroxyapatite nanoparticles demonstrated a 6.8-fold increase in bone targeting compared to conventional formulations (270), while pH-responsive lipid-based systems improved DKK1 gene silencing efficiency by 3.2-fold (271). Folic acid-modified paclitaxel liposomes exhibited a 5.3-fold higher accumulation in metastatic foci (272). Moreover, biomimetic red blood cell membrane-coated nanoparticles achieved prolonged circulation (t1/2 = 38 h) and, upon monthly prophylactic administration, improved micrometastatic clearance rates by 4.2-fold (273).

Epigenetic intervention represents a powerful preventive modality. Low-dose decitabine (0.1 mg/kg weekly) restores SFRP2 expression through demethylation, resulting in a 68% reduction in Wnt pathway activity (110). Natural compounds such as resveratrol, activating SIRT1 and repressing H3K4me3 histone modifications, have been shown to lower the five-year cumulative incidence of metastasis by 55% in high-risk populations (274).

Radionuclide therapy is emerging as a promising preventive strategy. 223Ra combined with olaparib extended median OS to 19.5 months (275), while 177Lu-PSMA-617 reduced mortality risk by 38% (276). α-particle emitters like 225Ac-PSMA and the short-range emitter 212Pb display superior tumoricidal activity, particularly against micrometastases (277, 278).

Molecular early warning and intervention strategies, such as PSMA-PET/CT using PROMISE V2 standards, achieve a sensitivity of 98% for detecting lesions smaller than 5 mm (279, 280). Gene therapy with BMP7 to induce tumor cell dormancy via p38MAPK activation yielded a dormancy maintenance rate of 68% (281, 282), while concurrent CXCR4 inhibition further eradicated residual dormant cells (265).

Finally, integration of therapeutic and preventive paradigms is paramount. Early administration of radium-223 (<3 months after ADT initiation) significantly improved bone metabolic markers (283). Emerging sequential regimens—neoadjuvant PSMA-RLT followed by radical surgery and adjuvant hormonal therapy—achieved a 15% pathological complete response rate (284). Targeting PSMA-negative resistant clones via multimodal PET imaging (PSMA/FDG/DOTATATE) and dual-ligand therapy restored sensitivity in 53% of cases (285). Artificial intelligence platforms, such as DeepMets (AUC = 0.93) and organoid-based drug sensitivity assays, are accelerating the individualization of metastasis prevention strategies (286, 287).

In summary, prevention of prostate cancer bone metastasis demands a multifaceted approach encompassing microenvironment modulation, dormancy maintenance, immune surveillance enhancement, precise molecular targeting, and epigenetic reprogramming. Future integration of real-time molecular diagnostics, nanoengineering, and AI-driven decision-making will enable a dynamic, individualized preventive landscape.

### 6.4 Prevention and treatment strategies for bone metastasis in breast cancer

Bone metastasis in breast cancer remains a major complication, significantly impacting patient quality of life and survival. A multi-faceted approach, encompassing both therapeutic and preventive strategies, is crucial in managing this condition.

#### 6.4.1 Bone-modifying agents

Bisphosphonates, particularly zoledronic acid, have demonstrated efficacy in reducing the risk of bone metastasis and increasing lumbar bone mineral density (BMD) (288). Another critical agent, denosumab, a third-generation RANKL inhibitor, has shown significant benefits by delaying the first skeletal-related event (SRE) by 4.3 months and improving overall survival (OS) in estrogen receptor-positive (ER+) breast cancer patients (175). Furthermore, novel drug delivery systems, such as liposomal formulations of zoledronic acid, have increased targeted drug delivery efficiency by 5-fold while reducing nephrotoxicity by 70% (179).

#### 6.4.2 Targeted signal pathway interventions

CDK4/6 inhibitors, such as palbociclib in combination with letrozole, have shown promise in extending progression-free survival (PFS) in bone metastatic breast cancer patients by 11 months (24.8 vs 13.8 months), with a 72% reduction in tartrate-resistant acid phosphatase-positive (TRAP+) cells[5]. The combination of everolimus and exemestane targeting the PI3K/AKT/mTOR pathway has prolonged progression-free survival to 31 months (vs. 13 months) and reduced serum CTX levels by 45% (163). Wnt pathway inhibition through DKN-01 monoclonal antibody in phase II trials has shown a 40% increase in bone metastasis lesion shrinkage (289).

#### 6.4.3 Immunotherapy innovations

PD-L1 inhibitors, such as atezolizumab in combination with chemotherapy, have demonstrated an overall response rate (ORR) of 29% in triple-negative breast cancer patients with bone metastasis, with the PD-L1+ subgroup showing improved OS of 25 months (290). CAR-T cell therapy targeting RANKL has shown promising results in preclinical models by reducing osteolytic lesions by 68% (291). Oncolytic virotherapy, such as T-VEC combined with radiotherapy, also induces a distant effect with a response rate of 33% in untreated lesions (292).

#### 6.4.4 Novel drug delivery systems

Nanoparticle-based drug delivery systems, such as hydroxyapatite nanoparticles loaded with doxorubicin, have been shown to increase drug concentration in bone metastases(5.8 times), while reducing cardiotoxicity by 72% (182). pH/redox-sensitive micelles (DOX@ALN-HA) exhibit a 90% drug release rate in the bone microenvironment, with a tumor inhibition rate of 78% (293). Magnetic nanoparticles used for local hyperthermia (52 °C) in conjunction with immunotherapy resulted in a 91% tumor ablation rate (294).

##### 6.4.4.1 Risk stratification and monitoring

FRAX(full name) assessment combined with TBS (trabecular bone score) is utilized to identify high-risk individuals, initiating preventive interventions for those with a T-score ≤ -2.0 or two additional risk factors (210). [^18^F]NaF-PET/CT offers 92% sensitivity, effectively detecting early bone metastases as small as <2 mm (208). Liquid biopsy monitoring, particularly CTC-based BMP2 expression, has shown predictive value for bone metastasis risk, with an AUC of 0.87 (210).

##### 6.4.4.2 Pharmacological prevention

Adjunctive treatment with bisphosphonates has been shown to reduce the risk of bone metastasis by 28% in postmenopausal women (RR = 0.72) (295). Denosumab (60 mg/6 months) used prophylactically has reduced clinical fractures by 50% (296). Supplementation with vitamin D (2000 IU/day) to maintain serum 25(OH)D levels above 40 ng/mL has been associated with a 29% reduction in bone metastasis risk (220).

##### 6.4.4.3 Endocrine therapy optimization

Selective estrogen receptor degraders (SERDs) like elacestrant maintain anti-metastatic activity even in CDK4/6 inhibitor-resistant models by downregulating the ERK/MAPK pathway and reversing resistance (297). Ovarian function suppression (OFS) combined with zoledronic acid (4 mg/6 months) stabilizes lumbar BMD, contrasting with a 5.4% decline in the control group (288).

##### 6.4.4.4 Lifestyle interventions

Vibration therapy combined with vitamin D supplementation has led to a 1.8% annual increase in BMD and a reduction of pain scores by 2.3 points (298). Resistance training (3 times/week) has improved lumbar BMD by 2.1% and reduced vertebral fracture risk by 23% (299).

##### 6.4.4.5 Novel preventive agents

Sclerostin inhibitors, such as romosozumab, have demonstrated a 47% increase in bone volume and a 35% reduction in circulating CTCs in preclinical models (300). The BCMA×CD3 bispecific antibody teclistamab has shown a 38% rate of osteolytic lesion healing (301), while the integrin αvβ3 inhibitor MK-0429 significantly reduced bone resorption by 72% (122).

Combined Treatment Sequential Regimens:Sequential therapy using a “bisphosphonate → denosumab → radium-223” strategy has extended the median SRE-free survival time to 28 months (302).

##### 6.4.4.6 Radiotherapy synergy

Stereotactic body radiotherapy (SBRT) combined with denosumab has achieved a 92% one-year local control rate, with pain relief occurring within 2.1 weeks (209).

Metabolic Intervention:The combination of glutaminase inhibitors like CB-839 with bisphosphonates has shown a 79% tumor inhibition rate in preclinical models by inhibiting osteoclast activity (303).

#### 6.4.5 Monitoring technology innovations

Imaging Techniques:^68^Ga-PSMA PET/CT has demonstrated 88% specificity, improving upon traditional bone scans by 40% (304). AI-Assisted Monitoring:Deep learning algorithms have increased the accuracy of predicting pathological fractures to 91%, outperforming the Mirels scoring system by 23% (305). Molecular Monitoring:ctDNA testing for PIK3CA mutations can predict bone progression 8.4 weeks in advance with an AUC of 0.87, offering earlier intervention opportunities (216). The comparative strategies for preventing bone metastasis of endocrine cancer discussed in this chapter are summarized in **Table 2**.

**Table 2:** Comparative strategies for preventing bone metastases in endocrine cancers.

Category	Thyroid cancer	Neuroendocrine Neoplasms (NENs)	Prostate cancer	Breast cancer
Local Interventions	Stereotactic body radiation therapy (SBRT) for early-stage lesions (41, 113). Radiofrequency ablation with bone cementoplasty to prevent fractures.	No clear local intervention measures are mentioned; the core focus is on early detection (imaging + biochemical monitoring) and bone health preservation for prevention.	Mechanical stimulation (30 Hz) enhances osteocyte communication, reducing OPN secretion by 41% (264).	SBRT combined with denosumab achieves 92% one-year local control rate (209).
Systemic Therapies	Targeted therapies: lenvatinib, selpercatinib (234). Immune therapy enhances CD8+ T-cell infiltration (234) (238).Bone-modifying agents: bisphosphonates, denosumab (238).	1. Vitamin D supplementation: Cholecalciferol (1000–2000 IU/day) to maintain serum 25(OH)D > 30 ng/mL, preserving bone mineral density and potentially improving outcomes; 2. Bone-modifying agents (BMAs): Bisphosphonates and denosumab, considered for patients with multiple bone metastases (BMs), rapid tumor progression, or high-grade tumors; 3. For MEN1-related hyperparathyroidism: Parathyroidectomy or calcimimetics (e.g., cinacalcet) to mitigate bone loss and skeletal complications.	Denosumab reduces skeletal metastasis incidence by 42% (260). Bisphosphonates combined with TGF-β inhibitors Vitamin D3 maintains serum levels above 30 ng/mL (262).	Bisphosphonates reduce bone metastasis risk by 28% in postmenopausal women (295). Denosumab (60 mg/6 months) reduces clinical fractures by 50% Vitamin D supplementation (2000 IU/day) reduces bone metastasis risk by 29%.
Risk Stratification & Monitoring	Deep learning model integrating PET/CT, ctDNA mutations, and ALP levels (127). Liquid biopsy assays detecting miRNA combinations with 94% sensitivity.	1. Risk stratification: - Patients with liver or lung metastases have significantly increased BM risk (odds ratio [OR] = 32.98 and 35.78, respectively), thus ^68^Ga-DOTATATE PET/CT screening is recommended; - For high-grade (G2/G3) lung NECs: Machine learning models (e.g., gradient boosting, AUC 0.723) integrate clinical and metastatic factors for individualized risk prediction; SHapley Additive exPlanations (SHAP) analyses highlight liver metastasis, nodal stage, age, and sex as key predictors; - For hereditary syndromes (e.g., multiple endocrine neoplasia type 1 [MEN1]): Regular screening is vital (primary hyperparathyroidism accelerates bone loss and fracture risk); 2. Monitoring frequency: Routine patients: Every 3–6 months (imaging, biomarkers, and clinical evaluation); Patients with prior skeletal-related events (SREs): Closer monitoring every 2–3 months (median time to second SRE is 10 months); 3. Biomarkers: Chromogranin A (CgA, sensitivity up to 81%), Bone sialoprotein (BSP, correlates with BM presence and poor prognosis), bone-specific alkaline phosphatase (BSAP, better prognostic value), PINP/NTx (indicate bone metabolism),high-sensitivity C-reactive protein (hs-CRP) and ALP may signal early BM in unknown primary NEC.	Liquid biopsy detection of KLF4/RUNX2 ratios (AUC = 0.87) and exosomal miR-181a-5p levels (267).	FRAX assessment combined with TBS for high-risk identification (210) [^18^F]NaF-PET/CT detects early bone metastases <2 mm with 92% sensitivity (209). Liquid biopsy monitoring of CTC-based BMP2 expression (AUC = 0.87) (266, 267).
Molecular Targeting & Microenvironment Modulation	Denosumab (120 mg/month) reduces bone metastasis incidence (175, 241). TGF-β signaling inhibitors and histone deacetylase inhibitors reduce tumor burden and angiogenesis (129, 242–244). CXCR4 antagonists reduce MDSCs, enhancing CD8+ T-cell infiltration (306)	No clear molecular targeting and microenvironment modulation measures are mentioned in current data; future research aims to refine risk models by incorporating molecular data (e.g., Ki-67, TP53/RB1 mutations) and explore relevant regulatory strategies.	CXCR4 inhibitors like plerixafor eradicate dormant tumor cells. PARP inhibitors (e.g., olaparib) combined with abiraterone reduce progression risk by 34% (268). Neoantigen vaccines (NeoVax) achieve 82% five-year metastasis-free survival.	CDK4/6 inhibitors (e.g., palbociclib) extend PFS by 11 months. Everolimus and exemestane prolong PFS to 31 months Wnt pathway inhibition with DKN-01 increases lesion shrinkage by 40% (289).
Innovative Technologies	^68^Ga-PSMA PET/CT combined with 177Lu therapy for enhanced localization (126). Nanoparticle-based drug delivery systems improve drug concentration in bone lesions CRISPR/Cas9-mediated gene silencing reduces metastasis formation by 73% (124).	1. Imaging technologies: ^68^Ga-DOTATATE PET/CT: High sensitivity (85%–95%) for somatostatin receptor (SSTR)-positive BMs, outperforming bone scintigraphy and CT; Dual-tracer imaging (^68^Ga-DOTATATE + ^18^F-FDG PET/CT): Detects asymptomatic lesions, refines staging, and alters management in 12.2%–14.3% of high-grade (G2/G3) NEC patients; MRI: 90%–100% sensitivity for bone marrow infiltration and spinal cord involvement (especially in patients with unexplained anemia, thrombocytopenia, or elevated lactate dehydrogenase [LDH]); 2. Future direction: Exploration of novel tracers (e.g., SSTR5 ligands) to improve detection.	Hydroxyapatite nanoparticles increase bone targeting 6.8-fold (270) pH-responsive lipid-based systems improve DKK1 gene silencing efficiency by 3.2-fold. Biomimetic red blood cell membrane-coated nanoparticles improve micrometastatic clearance rates by 4.2-fold (273).	Nanoparticle-based delivery systems increase drug concentration in bone metastases by 5.8 times (182) pH/redox-sensitive micelles exhibit 90% drug release in bone microenvironment. Magnetic nanoparticles for local hyperthermia achieve 91% tumor ablation rate.
Preventive Frameworks	Three-level prevention system: Primary: TSH suppression therapy with bone-protective agents reduces incidence by 41% over five years (45, 95). Secondary: SBRT combined with targeted therapy improves PFS to 68% over two year. Tertiary: 223Ra and immune checkpoint inhibitors extend median survival by 4.3 months (115).	Core framework: “Early Detection - Risk Stratification - Bone Health Preservation - Lifestyle + Follow-Up Management”: 1. Early detection: Combination of advanced imaging (68Ga-DOTATATE PET/CT, MRI) and biochemical markers (CgA + BSP, etc., AUC > 0.75); 2. Risk stratification: Individualized screening protocols for high-risk populations (liver/lung metastasis, high-grade tumors, MEN1); 3. Bone health preservation: Vitamin D supplementation combined with BMAs (when necessary); 4. Lifestyle + follow-up: Adequate calcium/protein intake, low-impact exercise (e.g., walking, swimming), avoidance of bone trauma, regular monitoring and timely adjustment of preventive measures.	Early administration of radium-223 (<3 months after ADT initiation) improves bone metabolic markers (283). Sequential regimens (neoadjuvant PSMA-RLT → surgery → adjuvant hormonal therapy) achieve 15% pathological complete response rate (304).	Sequential therapy (“bisphosphonate → denosumab → radium-223”) extends median SRE-free survival to 28 months (302). Glutaminase inhibitors combined with bisphosphonates show 79% tumor inhibition rate (303).
Monitoring Innovations	Molecular imaging techniques (e.g., ^68^Ga-PSMA PET/CT) for enhanced tumor localization (304) biopsy assays for early detection of micro-metastasis.	1. Combined monitoring: ^68^Ga-DOTATATE PET/CT combined with biochemical markers (CgA, BSP) improves diagnostic accuracy (AUC > 0.75, sensitivity and specificity > 80%), 2. Risk prediction optimization: Machine learning models (e.g., gradient boosting, AUC 0.723) applied in high-grade lung NEC to integrate clinical factors, enhancing risk prediction and monitoring precision, 3. Dynamic adjustment: Elevated CgA or BSP may prompt early initiation of BMA therapy.	PSMA-PET/CT using PROMISE V2 standards detects lesions <5 mm with 98% sensitivity [254, 255]Gene therapy with BMP7 induces tumor cell dormancy with 68% maintenance rate (279, 280).	AI-assisted monitoring increases accuracy of predicting pathological fractures to 91% (305) ctDNA testing for PIK3CA mutations predicts bone progression 8.4 weeks in advance (AUC = 0.87) (266, 267)

SBRT, Stereotactic Body Radiation Therapy; TSH, Thyroid-Stimulating Hormone; PFS, Progression-Free Survival; ADT, Androgen Deprivation Therapy; PSMA, Prostate-Specific Membrane Antigen; SRE, Skeletal-Related Event; AI, Artificial Intelligence; ctDNA, Circulating Tumor DNA; CTC, Circulating Tumor Cell; miRNA, MicroRNA; MDSC, Myeloid-Derived Suppressor Cell; OPN, Osteopontin; PIK3CA, Phosphatidylinositol-4,5-Bisphosphate 3-Kinase Catalytic Subunit Alpha.

## 7 Conclusions and perspectives

Bone metastases from endocrine malignancies—encompassing thyroid carcinoma, neuroendocrine carcinoma (NEC), and endocrine-dependent cancers such as prostate and breast cancer—represent a major clinical challenge that severely impairs survival and quality of life. Despite recent advances in multimodal imaging, molecular profiling, and systemic therapy, early diagnosis and effective management remain hindered by tumor heterogeneity and the complex tumor–bone microenvironment.

### 7.1 Current progress and challenges

High-resolution imaging technologies, including *^68^Ga-DOTATATE PET/CT* in neuroendocrine neoplasms and *PSMA PET/CT* in prostate cancer, have dramatically enhanced diagnostic accuracy and staging precision. Similarly, the integration of biomarkers such as chromogranin A, bone-specific alkaline phosphatase (BSAP), and circulating tumor DNA (ctDNA) into diagnostic algorithms enables earlier and more specific detection of bone involvement. However, limited sensitivity in detecting micrometastases and the lack of standardized diagnostic workflows continue to restrict early intervention.

On the therapeutic front, targeted and systemic therapies have redefined disease control paradigms. Tyrosine kinase inhibitors (e.g., lenvatinib, apatinib) and selective RET inhibitors (e.g., selpercatinib) have extended progression-free survival in radioiodine-refractory thyroid cancer. In NECs, peptide receptor radionuclide therapy (PRRT) with has shown durable responses in somatostatin receptor–positive disease. For prostate and breast cancer bone metastases, combinations of or CDK4/6 inhibitors with bone-modifying agents (zoledronic acid, denosumab) have become standard, significantly reducing skeletal-related events. Despite these achievements, drug resistance, therapy-induced bone remodeling, and treatment-related toxicities remain unsolved barriers.

### 7.2 Perspectives

Future research must focus on integrated precision medicine that combines molecular diagnostics, computational modeling, and personalized therapy selection. Multi-omics profiling—spanning genomics, proteomics, and metabolomics—should be incorporated into clinical workflows to enable predictive stratification and early detection of bone metastases. Artificial intelligence (AI)-based radiomics models have shown promise in predicting metastatic potential and treatment response with over 90% accuracy. Furthermore, –based monitoring of circulating tumor cells (CTCs) and ctDNA could revolutionize surveillance and real-time therapy adjustment. Therapeutically, future directions emphasize microenvironment-targeted and immune-based interventions. Combination regimens integrating *immune checkpoint inhibitors* with bone-targeted agents or radiopharmaceuticals (e.g., radium-223) are being explored to overcome immune evasion and enhance local control. The development of and *bone-seeking radioligands* offers new strategies for improving drug specificity while minimizing systemic toxicity. Additionally, patient-centered multidisciplinary management—coordinating oncology, radiology, orthopedics, and nuclear medicine—remains essential for optimizing outcomes and preserving function.

In summary, the management of endocrine cancer bone metastases is entering a new era of molecular precision, multimodal integration, and immunologic innovation. Continued translational research, early clinical trial enrollment, and global collaboration will be pivotal to transforming bone metastasis from a terminal complication into a controllable chronic condition, ultimately improving both longevity and quality of life for patients.
